# Precise Editing of the *OsPYL9* Gene by RNA-Guided Cas9 Nuclease Confers Enhanced Drought Tolerance and Grain Yield in Rice (*Oryza sativa* L.) by Regulating Circadian Rhythm and Abiotic Stress Responsive Proteins

**DOI:** 10.3390/ijms21217854

**Published:** 2020-10-23

**Authors:** Babar Usman, Gul Nawaz, Neng Zhao, Shanyue Liao, Yaoguang Liu, Rongbai Li

**Affiliations:** 1 College of Agriculture, State Key Laboratory for Conservation and Utilization of Subtropical Agro-Bioresources, Guangxi University, Nanning 530004, China; babarusman119@gmail.com (B.U.); gulnawazmalik@yahoo.com (G.N.); nengzhao@st.gxu.edu.cn (N.Z.); 1817303014@st.gxu.edu.cn (S.L.); 2 State Key Laboratory for Conservation and Utilization of Subtropical Agricultural Bioresources, South China Agricultural University, Guangzhou 510642, China

**Keywords:** rice, CRISPR/Cas9, mutant, drought tolerance, proteins, circadian rhythm

## Abstract

Abscisic acid (ABA) is involved in regulating drought tolerance, and pyrabactin resistance-like (PYL) proteins are known as ABA receptors. To elucidate the role of one of the ABA receptors in rice, *OsPYL9* was mutagenized through CRISPR/Cas9 in rice. Homozygous and heterozygous mutant plants lacking any off-targets and T-DNA were screened based on site-specific sequencing and used for morpho-physiological, molecular, and proteomic analysis. Mutant lines appear to accumulate higher ABA, antioxidant activities, chlorophyll content, leaf cuticular wax, and survival rate, whereas a lower malondialdehyde level, stomatal conductance, transpiration rate, and vascular bundles occur under stress conditions. Proteomic analysis found a total of 324 differentially expressed proteins (DEPs), out of which 184 and 140 were up and downregulated, respectively. The *OsPYL9* mutants showed an increase in grain yield under both drought and well watered field conditions. Most of the DEPs related to circadian clock rhythm, drought response, and reactive oxygen species were upregulated in the mutant plants. Kyoto Encyclopedia of Genes and Genomes (KEGG) analysis revealed that DEPs were only involved in circadian rhythm and Gene Ontology (GO) analysis showed that most of the DEPs were involved in response to abiotic stimulus, and abscisic acid-activated signaling pathways. Protein GIGANTEA, Adagio-like, and Pseudo-response regulator proteins showed higher interaction in protein–protein interaction (PPI) network. Thus, the overall results showed that CRISPR/Cas9-generated *OsPYL9* mutants have potential to improve both drought tolerance and the yield of rice. Furthermore, global proteome analysis provides new potential biomarkers and understandings of the molecular mechanism of rice drought tolerance.

## 1. Introduction

Rice is an important staple food for developing countries, but its sustainable production is facing issues, including a lack of supply such as labor shortage, climate changes, land, as well as the rising cost of production and water [1,2]. Drought stress conditions affect plant growth at any stage as a result of reducing crop yield worldwide. The co-occurrence of different abiotic stresses during plant growth is due to the abrupt changes in climate and a shortage of fresh water supply which results in a reduction of overall crop yield. The employment of approaches based on the latest advances in genetic and genomics research is helpful to meet the demands of global food security in the aspect of environmental challenges [3,4].

Among the different stresses, drought remains the single most important factor limiting crop production worldwide and it has been predicted that more than half of the world’s cultivated land will face water scarcity in the year 2050 [5,6]. In a water-limiting environment, plants undergo a cascade of biochemical, molecular, physiological, developmental, and morphological changes [3,5]. Genetic study has revealed that drought tolerance is controlled by many genes, and some drought tolerance genes, such as DREB and AREB/ABF TFs, have been discovered [3,7]. In corn, molecular marker-assisted breeding and precision phenotyping have led to enhanced drought tolerance [8,9]. However, breeding efficiency and marker accuracy remain challenging [10]. Some progress has been achieved by utilizing transgenic approaches to boost drought tolerance in crops [3,7,11]. Many lab studies and field trials have shown that the transgenic expression of stress-perceptive genes resulted in enhanced abiotic stress tolerance [12,13]. The transgenic approaches are recently the mainstream method to bioengineer drought tolerance in crop plants.

The clustered regularly interspaced short palindromic repeats (CRISPR)-Cas9 (CRISPR-associated protein 9) genome editing technology is of great interest and has demonstrated great promise for rapidly addressing evolving challenges in crop cultivation [14,15]. CRISPR/Cas9 can be used to precisely edit target genes to achieve the desired mutations and has higher mutagenesis efficiency and significance than other gene editing technologies [1,14]. CRISPR-Cas9 technology has been vastly used and made remarkable progress in the plant genome editing for improving agronomic traits, plant protection, grain yield, and abiotic stress tolerance [1,6,14,15,16,17,18,19,20,21,22,23]. Recently, CRISPR-Cas9-guided *SRL1* and *SRL2* mutagenesis resulted in improved drought tolerance in rice at the seedling and mature stage [13]. *Arabidopsis OsT2/AHA1* novel mutants generated by CRISPR-Cas9 displayed increased stomatal responses [24]. CRISPRCas9-directed *ARGOS8* mutations resulted in improved grain yield under drought conditions in maize, revealing the potential of CRISPR/Cas9 to generate drought-tolerant crops by creating novel allelic variations in plant genome [25].

Molecular databases have long been a powerful tool to study the proteome-wide induced changes by mutations. The iTRAQ (isobaric tags for relative and absolute quantitation) have been broadly used for quantitative proteomics which is compatible with high speed and high-throughput and helpful to quantitatively analyze protein abundance [26]. To understand the regulatory mechanism of different proteins, the use of bioinformatics data to establish protein–protein interaction (PPI) networks of differentially expressed proteins (DEPs) leads to an improved understanding of the particular response of an organism at the whole proteome level. To clarify the changes triggered by the mutations in a plant genome, function prediction, and the identification of DEPs is a useful strategy. Gene Ontology (GO) and Kyoto Encyclopedia of Genes and Genomes (KEGG) analysis are helpful to elucidate the biological roles of DEPs and their response to different pathways [27,28].

Abscisic acid (ABA) plays pivotal roles in the plant developmental processes and abiotic stresses including salinity, drought, high and low temperature [29]. Modifications in the key enzymes responsible for ABA biosynthetic pathways showed improved plant growth and drought tolerance. During abiotic stress, ABA levels increased in the plant and an ABA receptor family, pyrabactin resistant-like/regulatory components of ABA receptors (*PYL*/*RCAR*), promotes the interaction between PYL and protein phosphatase 2C (PP2C). The PYL and PP2C interaction promotes the expression of ABA-responsive genes by releasing SNF1-related protein kinase (SnRK)) by PP2C repression. Then, an ABA-mediated signal is generated, which enables the plants for the acquisition of abiotic stress tolerance [29,30,31,32]. The overexpression of *AtPYL5* confers enhanced drought tolerance and leads to ABA hypersensitivity at the seedling stage [32]. In rice, 10-13 PYL family genes have been identified through bioinformatic approaches [33,34,35]. Recombinant technologies were used to characterize *OsPYL1*, *2*, *3*, *6*, *10*, *11*, and *12*, and found that *OsPYL1*, *2*, and *3* are dimers [34]. It was found that the overexpression of *OsRCAR5*/*OsPYL5* resulted in ABA hypersensitive germination, plant growth, and enhanced drought and salt tolerance but reduced plant height and grain yield under well watered conditions [36]. The overexpression of *OsPYL3* and *OsPYL9* resulted in ABA-hypersensitivity during germination and tolerance to cold and drought stress at the seedling stage [35]. *OsPYL9* is located on chromosome 6 and shares 92% identity in amino acid sequence with *OsPYL8* and acts as a positive regulator of the ABA signaling pathway [37]. To date, different efforts have been being made to unveil the PYL gene functions but their CRISPR/Cas9 knockout mutants have not been generated and evaluated.

Many tools have been built to investigate the plant response towards different abiotic stresses but the vastly complex mechanism underlying the cell’s response to targeted mutations is still to be understood. Several studies designed to study the rice drought-tolerant mutants, but to date, there is no study available in which combined CRISPR/Cas9 and iTRAQ-based proteomic analysis has been performed to study the *OsPYL9* rice drought-tolerant mutants. In this follow-up study, we used CRISPR/Cas9 technique to generate *OsPYL9* loss-of-function mutants. Furthermore, the proteomic analysis of mutants and wild type (WT) was performed to assess the alteration at the whole proteome level. We presented the new results and defined how mutations may have impacted the expression of proteins at the global level. In this study, the CRISPR mutants showed enhanced drought tolerance under drought stress and improved grain yield under both normal and water-deficit conditions. Multiple identified proteins were differently expressed, and most of the DEPs related to circadian clock rhythm, drought response, and antioxidant activities were upregulated in the mutants. The results of the present study might lay a practical foundation for genetic and molecular mechanisms and the development of drought-tolerant and high yielding rice lines.

## 2. Results

### 2.1. Assembly of Targets in Vector

**Figure 1:**
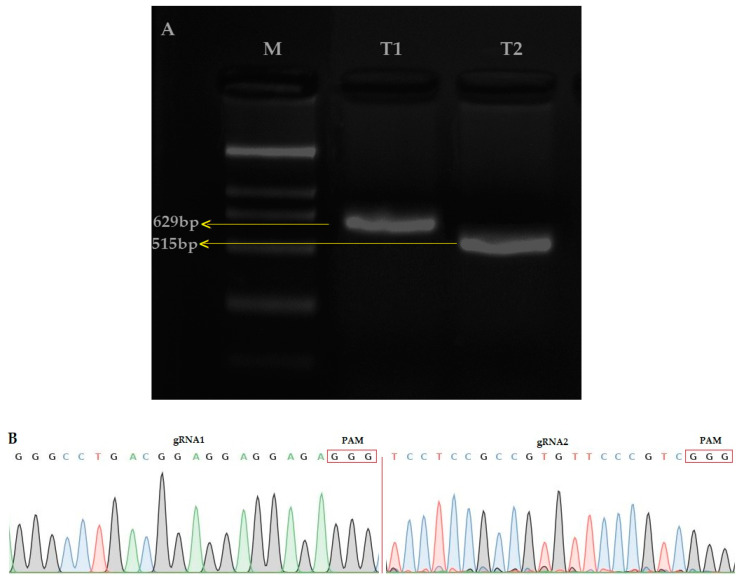
(**A**) Single guided RNA (sgRNA) expression cassette after the second round of PCR, M: 2000, Table 1. (OsU6a-gRNA): 629 bp, T2 (OsU6b-sgRNA): 564 bp (**B**) Sequencing peak map of both target sites (Target 1 and Target 2), assembled in vector.

The amplification of the single guided RNA (sgRNA) expression cassette for the first and second target (T1 and T2) was verified by the overlapping polymerase chain reaction (PCR) (T1; 629bp and T2; 515) (Figure 1A). The CRISPR/Cas9 binary vector was effectively built and both sgRNA sequences were confirmed in the vector (Figure 1B) by using the SP-L1 and SP-R (Supplementary Materials Table S1) primers. The results showed that the two target sequences assembled through the *Bsa* I site were consistent with the designed target sequence, so the constructed pYLCRISPR/Cas9 vector was considered suitable for the Agrobacterium-mediated rice genetic transformation.

### 2.2. Editing of OsPYL9 and Analysis of Unmarked T_0_ Generation

**Figure 2:**
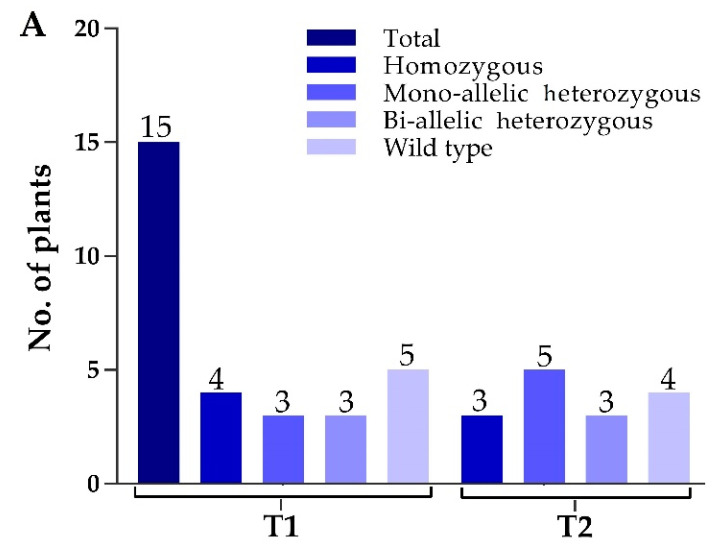

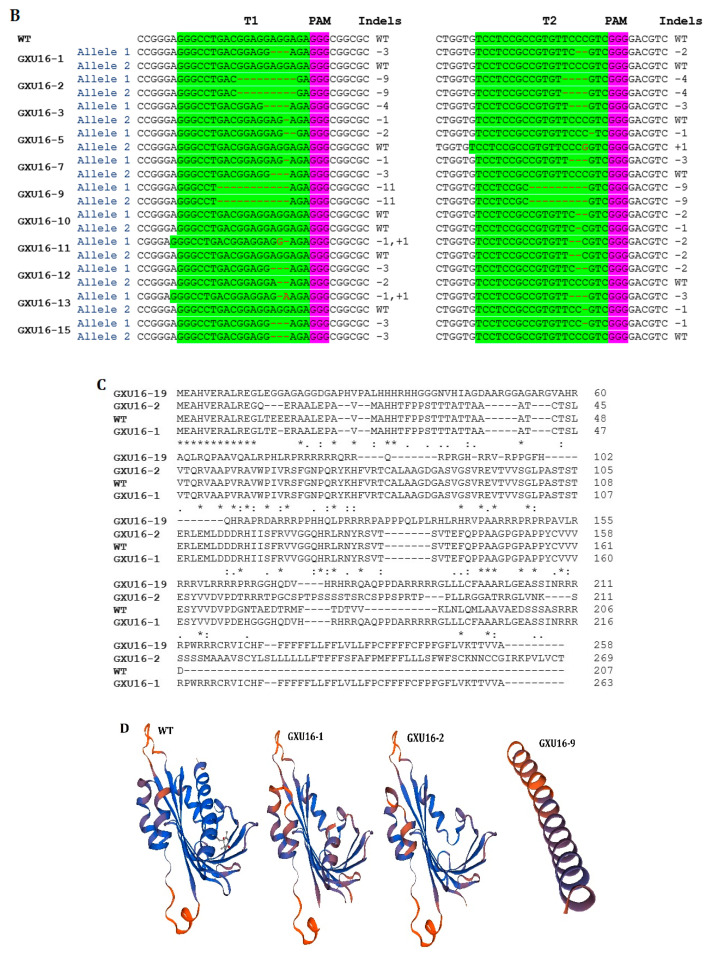
Mutation frequency, DNA, and amino acid sequence alignment of T_0_ mutant plants. (**A**) The number of total and mutant plants obtained in T_0_ generation; (**B**) DNA sequence alignment for wild type (WT) and mutant plants. Base deletion and insertion are represented with “-“ and red letters, respectively. Green and purple highlighted are the target regions and protospacer adjacent motif (PAM) sequence, respectively; (**C**) amino-acid sequence alignment and structure modeling of WT and mutant lines (GXU16-1, GXU16-2, and GXU16-9) in T_0_ generation. The asterisk “*” sign showed conserved amino-acid sequence regions. The deleted amino acids are shown by black hyphens. The multiple sequence alignment was performed using the Clustal Omega Multiple Sequence Alignment (https://www.ebi.ac.uk/Tools/msa/clustalo/); (**D**) the three-dimensional structures of the WT protein and its mutant lines (GXU16-1, GXU16-2, and GXU16-9).

Two constructs were transformed into IR-96, analyzed, and tested. In total, we treated 60 calli with transformed *A. tumefaciens* and attained 15 rice seedlings. We extracted the corresponding genomic DNA from each mutant plant, and target-specific primers were used to amplify the target regions to verify the mutations. The sequencing results displayed that the CRISPR/Cas9 vector was successfully inserted into the plant genome, and 11 independent mutant lines were edited near the protospacer adjacent motif (PAM) region, which represents an editing efficiency of 73.33% (Figure 2A). By comparing *OsPYL9* reference sequence of WT with mutant plants, the genome editing patterns of T_0_ edited lines resulted in homozygous and mono-allelic and bi-allelic heterozygous mutations. There were four homozygous, three mono-allelic heterozygous, three bi-allelic heterozygous, and five WT plants at the first target and three homozygous, five mono-allelic heterozygous, three bi-allelic heterozygous, and four WT plants at the second target. Two mutant lines (GXU16-2 and GXU16-9) showed homozygous mutations for both target sites (Table S2). GXU16-2 exhibited 9 bp and 4 bp deletions at the first and second target positions, respectively. GXU16-9 presented 11 bp and 9 bp deletions at the first and second target locations, respectively (Figure 2B). Mutations such as the deletion, insertion, or substitution of at least one nucleotide were achieved, which caused an in translation shift, the early termination of the coding sequence, and/or the deletion of amino acid residue.

Various types of mutations were found with deletions, simultaneous deletions, and insertions while there was no mutation with only insertions (Table S2). The 1bp deletions ranked first with 47.6% in the first target and 33.33% in the second target. Most of the mutations were short-ranged 1–4 bp, although there were also longer deletions of 10 bp and 11 bp. We selected GXU16-1 (mono-allelic heterozygous), GXU16-2 (homozygous), and GXU16-9 (homozygous) lines for amino-acid sequence alignment and modeling of protein structure. Mutant lines showed changed amino-acid sequence (Figure 2C). Modeling of protein structures showed a significant difference in WT and mutant lines, whereas the homozygous mutant line showed a more different and a shortened protein structure (Figure 2D). Using the specific primers, we amplified the DNA of 35 T_1_ mutant plants (Table S3) for the five most likely positions with the maximum ranking off-target potential. The sequencing results revealed that there were no off-target effects found in selected putative loci against sgRNA1 and sgRNA2 (Table S4).

### 2.3. Screening of Transgene-Free Plants and Segregation Analysis in T_1_ Generation

To screen the transgene (T-DNA) free genome edited rice plants, the T_1_ generation was evaluated. We screened a total of 15 mutant plants to detect the absence or presence of the exogenous DNA of the mutant lines by using cas9-specific primers cas9-F/Cas9-R and HPT-F/HPT-R primers (Table S1). The fragmentation and truncations of Cas9 or HPT could be easily missed using only simple PCR. We used proteomic analysis to confirm if transgene expression was absent or present at the protein level. The results showed that five mutant plants (GXU16-4-1, GXU16-6-1, GXU16-12-1, GXU16-13-1, and GXU16-15-1) were amplified to the Cas9 vector sequence and the HPT primers, while GXU16-10-1 was only amplified to the HPT primers, with 572 and 672 bp fragment length, respectively (Figure S1). We confirmed that the Cas9-protein or HPT-protein was detected only in the plants which showed bands for Cas9- or HPT-specific primers, respectively (Supplementary file 2). Those plants were termed as T-DNA-free which do not amplify to the corresponding fragment or their abundance was not detected in the proteomic experiment. In total, 60% of the plants appeared to be T-DNA-free. Based on *these results*, it was concluded that the designed *primers* were efficient to screen the T-DNA-free plants. The progeny of T_0_ generation was evaluated for segregation analysis. The homozygous mutations showed the same mutation type, whereas mono-allelic and bi-allelic heterozygous mutants followed the classic Mendelian inheritance pattern (1:2:1) (Table S5).

### 2.4. Agronomic Traits Evaluation under Normal and Drought Conditions

We selected the GXU16-1, GXU16-2, and GXU16-9 mutant lines for further investigations. The mean results for the agronomic traits in T_0_ generation showed that some of the characters showed a significant difference between the mutant lines and WT. Under normal watering conditions, mutant lines showed significantly increased 1000-grain weight (GWT), grain length (GL), grain width (GWD), and yield per plant (YPP), and decreased flag leaf length (FLL), and flag leaf width (FLW), whereas, there was no change in plant height (PH), panicle number (PN), and panicle length (PL) under normal watering conditions. Under the water deficit conditions, mutant plants showed increased PH and PL, grain number per panicle (GNPP), GWT, GL, GWD, and YPP, whereas decreased FLL and FLW under drought conditions (Table 1). The FLL and FLW were decreased from 53.1 to 38.5 cm, and 2.2 to 1.5 cm, respectively; the GWT was increased from 29.5 to 38.7 g; the GL and GWD of the mutant lines were increased from 9.0 to 11.3 mm, and 29.5 to 38.7 mm, respectively; and the YPP was increased from 30.8 to 40.6 g.

We recorded the data for main agronomic traits to investigate the effects of *OsPYL9* mutations also in T_1_ generations. As shown in Table 1, CRISPR mutants significantly increased GWT, GL, GWD, and YPP, whereas they decreased FLL and FLW under normal conditions. Homozygous mutants showed increased grain yield also under drought stress conditions a little more than the heterozygous plants. The results were consistent with T_0_ generation, which clearly showed that mutations were passed to the next generation successfully.

### 2.5. The Effect of OsPYL9 Mutagenesis on Abscisic Acid (ABA), Malondialdehyde (MDA) and Enzymatic Activities under Normal Conditions and Drought Stress

**Figure 3:**
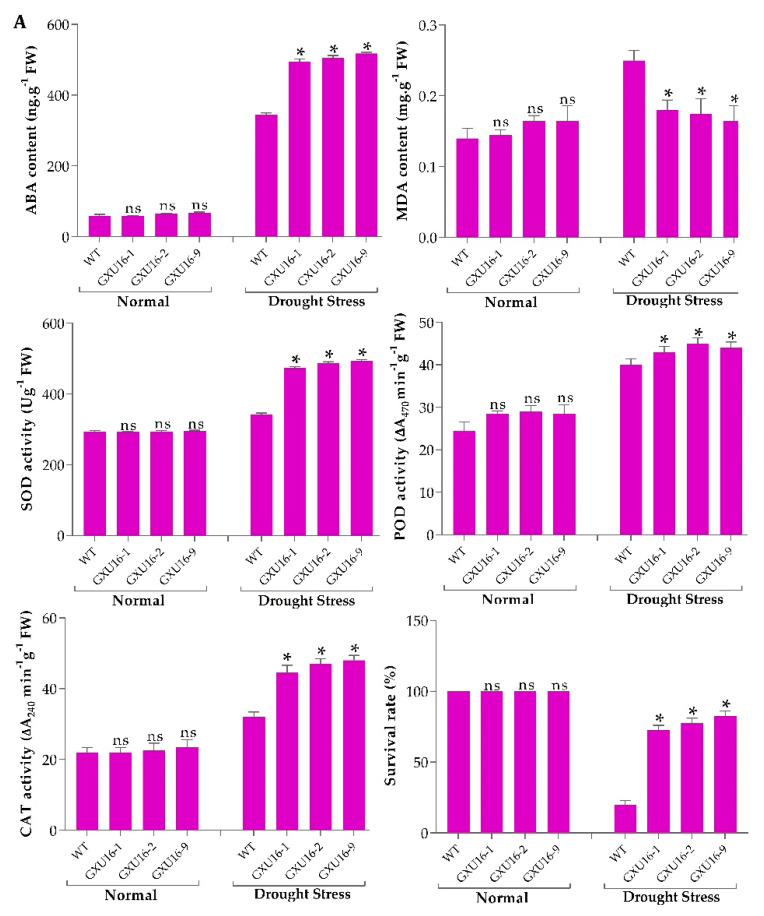

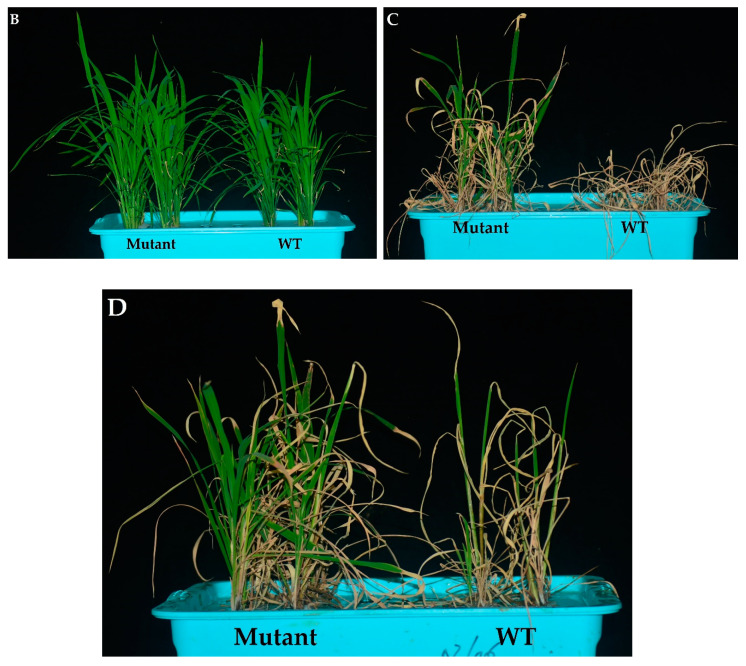
(**A**) Effects of *OsPYL9* mutations on abscisic acid (ABA), malondialdehyde (MDA) levels, enzymatic activities, and the survival rate of WT and mutant plants under normal and drought stress conditions. (**B**) Phenotype of WT and mutant plants at the seedling stage before drought stress, (**C**) under drought stress, and (**D**) after re-watering. WT plants showed non-significant and significant differences under normal and drought conditions, respectively. Data are presented as the means ± SD (*n* = 5). * and ^ns^ indicate a significant and non-significant difference, respectively, Student’s *t*-test, *p* ≤ 0.01.

We investigated ABA and malondialdehyde (MDA) levels in the WT and *mutant* plants at the seedling stage. There was no significant difference observed in the ABA or MDA levels under normal conditions, whereas a 50.43% increase in ABA levels was observed in mutant lines as compared to WT (Figure 3A) under drought stress. Under drought stress, the MDA level was significantly increased in WT plants, as compared to the mutant lines (Figure 3A). Among the mutant lines, homozygous lines GXU16-2 and GXU16-9 had higher ABA levels while heterozygous lines GXU16-1 had slightly lower ABA levels under drought stress. These results showed that homozygous mutants accumulated more ABA as compared to heterozygous plants under drought stress and thus were more tolerant of dehydration. These findings suggest that *OsPYL9* may play an important role in ABA and MDA signaling.

Under normal conditions, the antioxidant activities did not differ in WT and mutant plants at the seedling stage. However, superoxide dismutase (SOD), peroxidase (POD), catalase (CAT) activities were higher under drought conditions and mutant lines showed significantly increased enzymatic activities as compared to WT plants (Figure 3A). The drought tolerance assay showed that the survival rate of WT plants was 20%, while 82.5% of mutant plants were recovered. Before drought stress, WT and mutant plants showed a similar phenotype at the seedling stage under normal conditions (Figure 3B). Mutant plants showed green leaves under drought stress, while the WT plants were wilted. WT plants showed more physiological damage under drought stress (Figure 3C), and mutant plants showed a green phenotype and complete recovery after re-watering (Figure 3D).

### 2.6. Measurement of Chlorophyll Content, Transpiration Rate, and Stomatal Conductance

**Figure 4:**
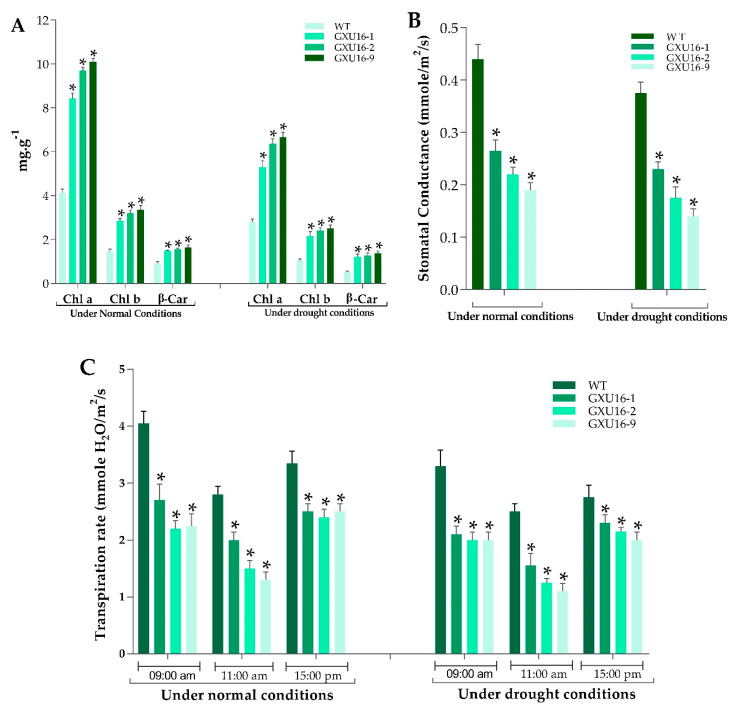
Performance of WT and *OsPYL9* mutants for (**A**) chlorophyll content (**B**) stomatal conductance and (**C**) transpiration rate. WT plants showed a significant difference under both normal and drought conditions. Data are presented as the means ± SD (*n* = 5). * indicate a significant difference, Student’s *t*-test, *p* ≤ 0.01.

As shown in Figure 4, the chlorophyll a (Chl) a and chlorophyll b (Chl b) contents of mutant plants were significantly increased as compared to WT, under normal and drought conditions. Under normal conditions, the mutant lines GXU16-1, GXU16-2, and GXU16-9 showed Chl a content of 8.26, 9.81, and 9.99 mg·g ^−1^, respectively, while the WT showed 4.25 mg·g ^−1^ Chl a content. The Chl b content in WT was 1.54 mg·g ^−1^ and for the mutant lines GXU16-1, GXU16-2, and GXU16-9, we found 2.81, 3.12, and 3.2 mg·g ^−1^ of Chl b content, respectively. β-carotene contents of mutant lines GXU16-1, GXU16-2, and GXU16-9 were 1.47, 1.52, 1.54 mg·g ^−1^ fresh weight, respectively, and for WT were 0.97 mg·g ^−1^. Under drought conditions, Chl a and Chl b contents were significantly increased from 2.91 to 6.52 mg·g ^−1^ and from 1.1 to 2.4 mg·g ^−1^, respectively. The β-carotene contents were also significantly increased from 0.49 to 1.3 mg·g ^−1^ (Figure 4A). Physical limitations associated with having a reduced stomatal density were estimated by calculating the stomatal conductance. The mutant plants showed significantly lower stomatal conductance than WT under normal and drought conditions (Figure 4B). Significant variation in transpiration was observed in WT and mutant plants under normal and water stress conditions. Notably, the transpiration rate of mutant plants during sunny days was lower at the three different time points (Figure 4C).

### 2.7. OsPYL9 Mutant Accumulated More Waxy Crystals on the Leaf Epidermis and Showed Decreased Vascular Bundles

**Figure 5:**
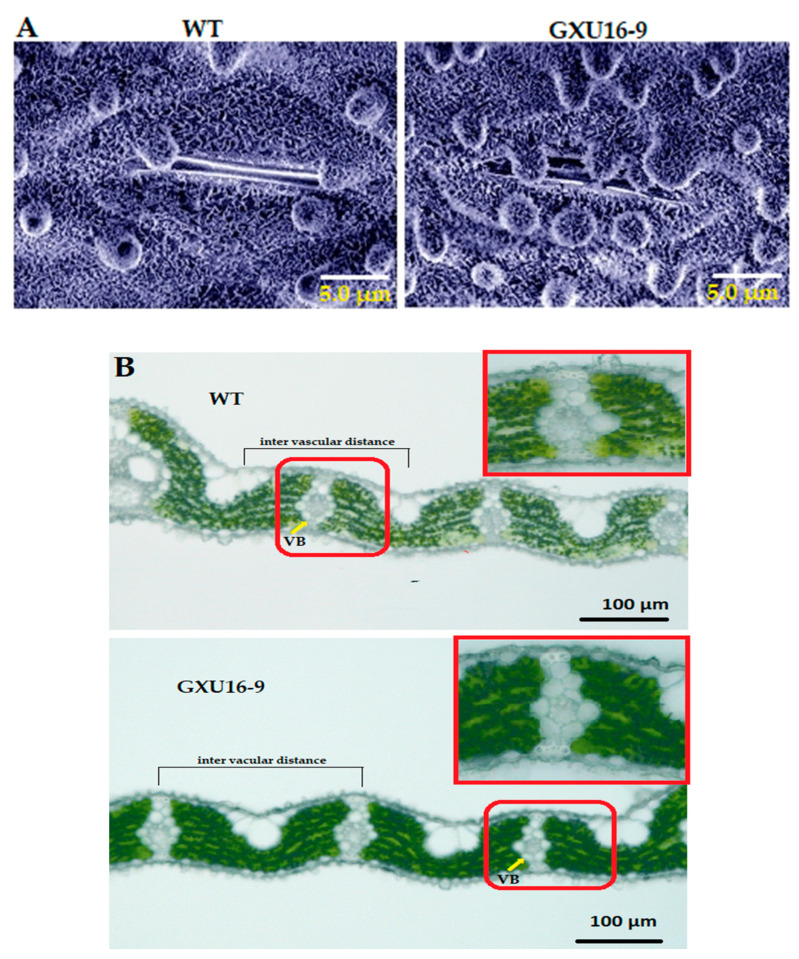
Microscopic analysis of wild type (WT) and mutant line GXU16-9 leaf surfaces. Scanning electron microscope (SEM) images of cuticular wax on adaxial leaf surface (**A**) and number and distance of vascular bundles (**B**) of WT and mutant line GXU16-9.

Adaxial leaf surfaces of WT and homozygous mutant line GXU16-9 were observed by scanning electron microscopy. Interestingly, platelet-like wax crystals deposited on leaves were larger in the mutant line’s leaf compared with WT. Results showed that the leaf surfaces of the mutant line were covered with a dense layer of wax crystals, including the unevenly distributed cuticular papillae, whereas fewer wax crystals and cuticular papillae around the stomatal apparatus were observed on the leaf surfaces of WT (Figure 5A). These results suggest that mutations in the *OsPYL9* effect saw the wax accumulation on the leaf surface. Moreover, the results showed that the WT leaf structure had a higher distribution density of vascular bundles and developed vascular bundle tissue. The distance between the vascular bundles of the WT was 208.5 ± 3.5 µm, which was slightly smaller than mutant line 226.6 ± 4.3 µm. The size of the vascular bundles in the WT leaves was larger than the mutant line, and the xylem was more developed, which was more conducive to water transportation (Figure 5B).

### 2.8. Peptide/Protein Identification and Absolute Quantitation

A total of 441,569 spectra were generated from the iTRAQ experiment for three independent replicates of WT and GXU16-9. We identified 91,115 known spectra, 34,858 peptides, 8815 proteins, and 5911 protein groups after analyzing the obtained spectra (Figure S2A). The protein mass distribution was outlined in Figure S2B. Proteins with 11–70 kDa accounted for more protein numbers and proteins with 1–10 kDa and 71–150 kDa showed a less protein number. Figure S2C showed the numbers of peptides identified in the proteins. Results revealed that identified proteins showed 30 peptides, and with the increase in peptides, the protein quantity was decreased. The peptide 1–2 contained maximum proteins (2264) and peptide 29–30 contained the least number of proteins (17). Most of the peptide’s lengths were around 1000–3858, and the number of peptides lengths of more than 17 were considered to be relatively low (Figure S2D). Additionally, 3416 proteins showed sequence coverage from 1–40% while only 611 proteins showed sequence coverage of more than 40% (Figure S2E). The error found in the database was below than 0.05 Da in the matching of the peptide segment (Figure S2F). The detailed information of all identified peptides and proteins is listed in Supplementary File 2.

### 2.9. Screening of Differentially Expressed Proteins (DEPs) and Most Enriched Pathways

To perform a comparison in a pairwise manner between WT and GXU16-9, both samples were firstly grouped. A scatter plot of all the expressed protein abundance and distribution was drawn to represent the distribution of DEPs in screening threshold proportions (Figure S3). Differential analysis between WT versus GXU16-9 showed 324 DEPs, inhibited 140 proteins, and increased the expression of 184 proteins (Additional File 2). The proteins were screened with a corrected false discovery rate (FDR) by a fold change (FC) of ≥1.50 and ≤0.67 for the upregulated and downregulated DEPs (Student’s *t*-test, *p* ≤ 0.05) because the gene editing changed the abundance of proteins. We also manually searched among all the DEPs that were directly involved in abiotic stress tolerance and reactive oxygen species (ROS) scavenging. A significant difference was observed between WT and the mutant line. We found eight upregulated DEPs, i.e., Q9AWL7 (GIGANTEA), Q657D6 (early flowering gene), Q689G9 (Pseudo-response regulator 1), A0A0N7KPA9 (MYB family transcription factor), Q0D3B6 (Days to heading 7), Q2R2W1 (FLAVIN-BINDING, KELCH REPEAT, F-BOX 1), C6F1N5 (Pseudo-response regulator 59), and Q689G6 (Pseudo-response regulator 95) related to circadian clock rhythms in mutant line. As expected, ten DEPs, i.e., Q9FXQ3 (Calcium-dependent protein kinase 13), Q5JLS2 (CBL-interacting protein kinase 12), Q6ZKN0 (WRKY transcription factor 30), Q0JQF7 (AP2/EREBP transcription factor), Q0JHF1 (ABA-responsive element binding factor 1), Q10NE1 (SALT-AND DROUGHT-INDUCED RING FINGER 1), Q0IUZ3 (NAC protein), Q0DMY6 (DROUGHT AND SALT TOLERANCE), Q64MA1 (Dehydration-responsive element-binding protein 1A), and Q6IEN1 (WRKY transcription factor) related abiotic stress response were found to be upregulated in the mutant line. Moreover, we found some DEPs related to CAT (A3REN3), POD (B8ARU3, A2YPX2, A2X2T0, B8A755, B8B5W7, A2X822, B8B3L5, B8ASV8, A2Z4F1, B8B653, B8B5W6, Q01MI9, A2WPA1, A2WNR8, B8APG3, B8ARU4, A2Z9R2, B8BM92, and A2ZAQ7), and SOD (B8AWM4 and A2YY59) (Table 2).

The significantly enriched pathways (*p* ≤ 0.05) are presented in Table S6. The *p*-value for the enriched pathways was corrected for multiple testing. Results revealed that the DEPs were enriched in nine different pathways including ribosome biogenesis in eukaryotes, carotenoid biosynthesis, phenylpropanoid biosynthesis, cysteine, and methionine metabolism, starch and sucrose metabolism, phagosome, circadian rhythm, carbon metabolism, and homologous recombination pathway.

### 2.10. Functional Assignment of the Differentially Expressed Proteins (DEPs)

A set of DEPs was subjected to a GO and KEGG analysis and their different functions were identified. The significant enrichment analysis of DEPs GO function was clarified and the differences between the two samples at the functional level were identified. The *p*-value was adjusted by multiple hypothesis tests and the enriched GO terms were selected by FDR (false discovery rate).

**Figure 6:**
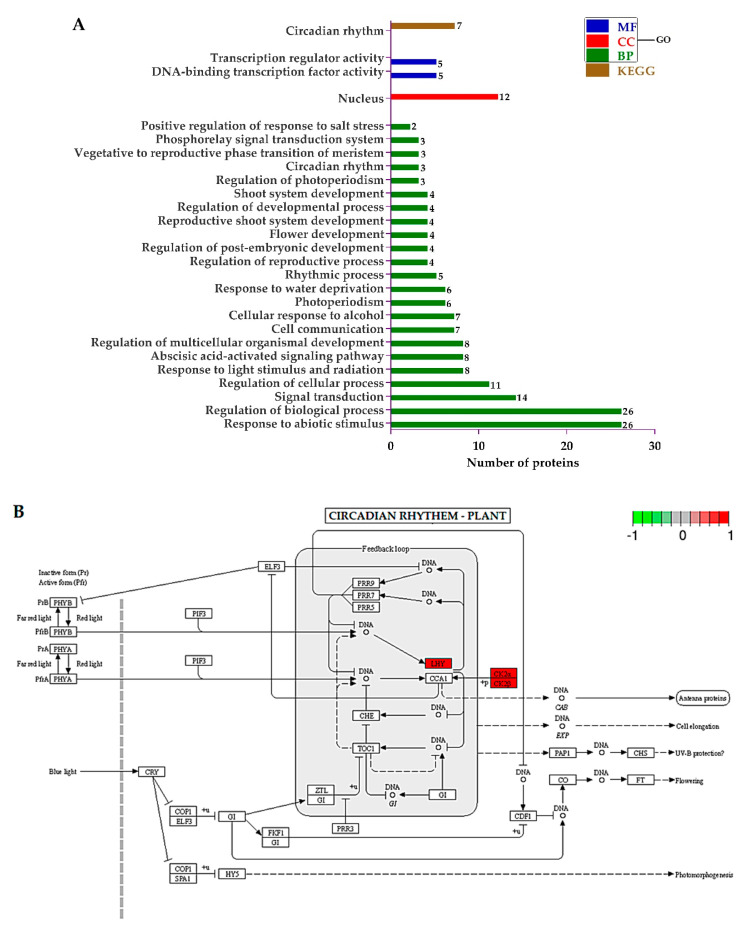
(**A**) Histogram showing the significantly enriched Gene Ontology (GO) annotations and Kyoto Encyclopedia of Genes and Genomes (KEGG) pathways of differentially expressed proteins (DEPs), and (**B**) Pathway built by Pathview. Red highlighted enzymes were upregulated.

The GO terms regarding “biological process (BP)”, showed that the DEPs related to the response to abiotic stimulus, the regulation of biological process, signal transduction, the regulation of cellular process, response to light stimulus and radiation, abscisic acid-activated signaling pathway, and the regulation of multicellular organismal development were significantly regulated. Regarding “cellular component (CC)”, only proteins associated with the nucleus were enriched. Finally, from the “molecular function (MF)” perspective, proteins involved in DNA-binding transcription factor activity and transcription regulator activity were sufficiently regulated. The KEGG pathways were only involved in circadian rhythm (Figure 6A). The KEGG pathway diagram rendered by Pathview showed that the late elongated hypocotyl (LHY) and two regulatory subunits (CK2α and CK2β) of casein kinase II were upregulated in mutant plants (Figure 6B).

### 2.11. Functional Interaction Networks of the Differentially Expressed Proteins

**Figure 7:**
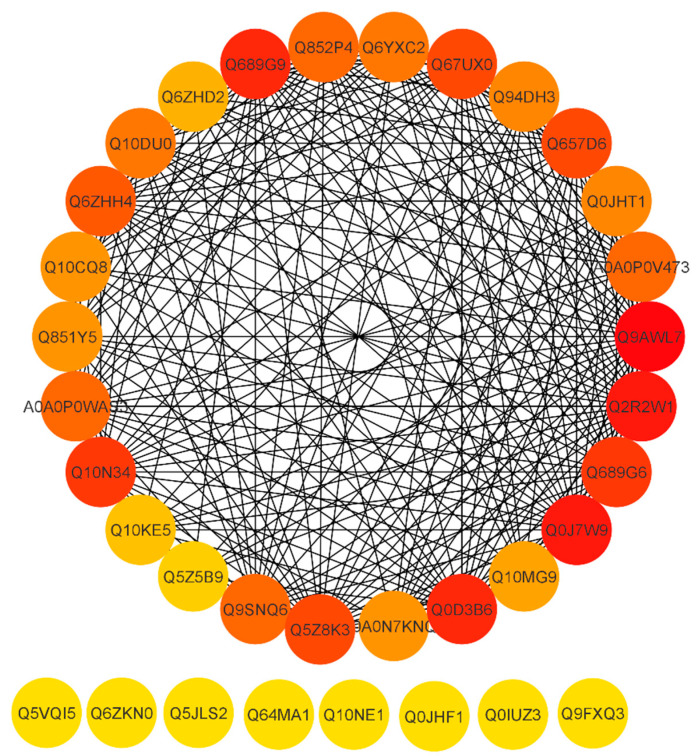
STRING software-predicted protein to protein network of differentially expressed proteins (DEPs). Differentially accumulated proteins are represented by a node, whereas the different color of lines represents evidence for the predicted functional relationship. The strong interaction is indicated by redder color. The proteins outside the circle showed no or weak interaction.

To reveal a cellular network of protein interactions, the Search Tool for the Retrieval of Interacting Genes/Proteins (STRING) database was used. Given a list of the proteins as input, STRING can search for their neighbor interactors, and the proteins that have direct interactions with the inputted proteins; then STRING can generate the PPI network consisting of all these proteins and all the interactions between them. The degree of a protein in a network is an important topological property, which indicates the number of partners it interacts with. Here, were we predicted the PPI network on the basis of degree. Essential proteins always have a high degree to form hubs in PPI networks. As a result, essential proteins appear to be the pivot in PPI networks and probably are associated with many fundamental biological processes. The proteins are represented by a node and the line between the nodes which indicates the PPI network. Highly connected nodes are central to a network’s architecture and function. Highly interacting proteins serve as a molecular signature to regulate the specific function. After extracting proteins with the highest connectivity from the predicted network, higher interaction was found between Q9AWL7 (Protein GIGANTEA), Q0J7W9 (Os08g0157600 protein), Q2R2W1 (Adagio-like protein 3), Q0D3B6 (Protein Days to heading 7, 2), Q689G9 (Pseudo-response regulator 1; OsPRR1), Q10N34 (Pseudo-response regulator 73; OsPRR73), Q689G6 (Pseudo-response regulator 95; OsPRR95), Q67UX0 (Putative adagio-like protein 2), Q5Z8K3 (Adagio-like protein 1), Q657D6 (ELF3-like protein 2), and Q6ZHH4 (E3 ubiquitin–protein ligase) (Figure 7) with a degree higher than 20 (Supplementary file 2). Some proteins including Q10NE1 (Zinc finger family protein), Q64MA1 (Dehydration-responsive element-binding protein 1A), Q5JLS2 (CBL-interacting protein kinase 12), Q6ZKN0 (WRKY30), Q5VQI5 (Os01g0165100), Q9FXQ3 (Calcium-dependent protein kinase 13), Q0IUZ3 (Os11g0126900), and Q0JHF1 (bZIP transcription factor 12) showed a degree value of 0 and no interaction with any other protein. The above results revealed that the pseudo-response regulator and adagio-like proteins were found to be highly interactive. Conclusions can be drawn that these proteins may be potential participants of the rice circadian rhythm and drought responsive pathways.

### 2.12. Quantitative Real-Time-qPCR-Based Assessment of OsPYL9 Expression Level and Validation of DEPs

**Figure 8:**
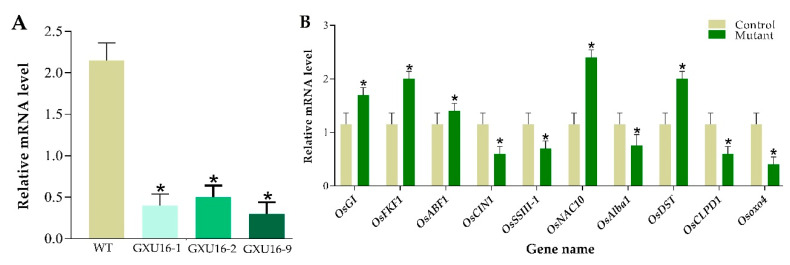
Real-time quantitative PCR validation of the *OsPYL9* expression level in wild type and mutant lines, and ten selected differentially expressed proteins (DEPs) responsive genes under normal conditions. (**A**) Expression analysis of *OsPYL9* in wild type (WT) and mutant lines (GXU16-1, GXU16-1, and GXU16-9). (**B**) Relative expression level of ten selected DEPs responsive genes. The data were analyzed by three independent repeats, and standard deviations were shown with error bars. Significant differences in the expression level were indicated by “*”, Student’s *t*-test, *p* ≤ 0.01.

Total RNA was isolated for the analysis of *OsPYL9* expression level in WT and transgenic plants by performing RT-qPCR. Rice *Actin* gene was used as a reference to normalize *SRL1* expression between samples and the results showed that the expression of *OsPYL9* was significantly suppressed in all mutant lines (Figure 8A). To validate the differential expression levels of some genes, the RT-qPCR assay was carried out for WT and mutant plants. In total, ten random genes were randomly selected with five downregulated proteins including Beta-fructofuranosidase, insoluble isoenzyme 1 (*OsCIN1*), Soluble starch synthase III-1 (*OsSSIII-1*), dehydration-responsive nuclear protein (*OsAlba1*), ATP-dependent Clp protease ATP-binding subunit (*OsCLPD1*), and Oxalate oxidase 4 (*Osoxo4*), and five upregulated proteins including, GIGANTEA (*OsGI*), FLAVIN-BINDING, KELCH REPEAT, F-BOX 1 (*OsFKF1*), ABA-responsive element binding factor 1 (*OsABF1*), NAC protein (*OsNAC10*), and DROUGHT AND SALT TOLERANCE (*OsDST*). The results clearly demonstrate that the expression pattern of these selected genes was consistent with the proteomic data (Figure 8B).

## 3. Discussion

Drought stress is the most serious environmental factor which impacts crop productivity worldwide. Our study demonstrates that *OsPYL9* is a drought sensitive gene and its mutations significantly increased the yield and drought tolerance in rice. This study opens a new avenue to understand the stress-sensitive mechanisms for improving plant abiotic stress tolerance. To our knowledge, this is the first report of increasing drought tolerance by targeting *OsPYL9* in rice using CRISPR technology. The CRISPR/Cas9 system has appeared as a powerful and latest tool for targeted gene mutations in crop plants [20,21,22,23]. In the present study, drought-tolerant rice mutant lines with increased yield were generated via CRISPR/Cas9, and proteomic analysis was performed for the characterization of mutant plants which may be useful for rice breeding.

Knockouts of the *OsPYL9* by CRISPR-Cas9 significantly increased grain yield under field drought and well watered conditions. These results show that the *OsPYL9* knockout rice lines improve drought tolerance and yield under well watered and severe drought stress conditions (Table 1). No abnormal visible phenotypes were observed with the CRISPR-generated lines during the rice growth and development stages under both drought and well watered field conditions. The consistent results of *OsPYL9* gene by CRISPR technologies under field conditions clearly demonstrated the feasibility of improving drought tolerance by editing rice genes.

Heritable homozygous mutations are highly desirable in molecular breeding [21]. According to the Sanger sequencing results of the T_0_ plants, they are both 24.08% homozygous, 44.82% heterozygous, while 31.10% were WTs. These results suggest the efficiency of the CRISPR/Cas9 system in rice and the homozygous mutants were reported in the T_0_ generation in previous reports [21,22]. There were no off-targets observed in the five most likely sites and T-DNA-free plants were screened with a frequency of 60%. Off-target events are rare in higher plants and important concerns in the application of CRISPR/Cas9 and previous studies also suggested that inherited Cas9 rarely induces off-target mutations and concluded that the off-target rate in rice is very low [23]. Selecting T-DNA-free plants might be effective to reduce the probability of off-target mutations [19]. In our results, most deletions occurred between 2bp and 3bp upstream to PAM.

Mutant plants showed increased chlorophyll content and decreased stomatal conductance and transpiration rate as compared to the WT under normal and drought stress conditions. Anatomical observations revealed that the *OsPYL9* mutant resulted in increased leaf cuticular wax content and decreased vascular bundles. Mutants showed a higher survival rate and antioxidant activities at the seedling stage and higher grain filling percentage at maturity. ABA is an important signal in response to drought stress and it can regulate the ROS generation [38]. In previous studies, mutations in the rice *OsCHR4* and microRNA166 induce narrow and rolled leaves with increased cuticular wax, smaller bulliform cells (BCs), and reduced stomatal conductance and transpiration resulting in improved drought tolerance in reduced water loss rate and enhanced drought tolerance [39,40].

In this work, the comparative iTRAQ-proteomics analysis was used to identify the differentially accumulated proteins in the WT and mutant plants. Protein extraction was performed from the fresh leaves of WT and mutant plants at the same stage. The protein concentration was measured by Bradford protein assay and an equal amount in each sample was confirmed for the normalization before further analysis. A total of 91,115 known spectra were produced and 8815 proteins were identified. A total of 324 DEPs were found between the WT and mutant plants. By comparison, we identified different DEPs involved in abiotic stress tolerance. To comprehend the underlying molecular interactions among different proteins, functional, pathway, and interaction analysis was performed.

Studies have revealed that variations in wax biosynthesis-related genes (*CER1*, *CER6*, *GL8*, and *WSL1,* *GL1*, *WAX2*, *OsGL1-1* (*WSL2*), *Wda1*, *WXP1*, *OsGL1-2,* and *WIN1*) affect cuticular wax accumulation and cuticle structure [41,42,43,44,45]. *LACS2* effect cuticle structure, however *WAX2*, *Wda1*, *GL1*, *OsGL1-1* (*WSL2*), *OsGL1-2, WIN1,* and *WXP1,* affect both cuticular wax accumulation and cuticle structure [46,47,48,49,50,51]. These results suggest that *OsPYL9* is involved in leaf cuticular wax accumulation. Cuticular waxes are complex mixtures of very-long-chain fatty acids (VLCFAs) and their derivatives which play an important role to prevent non-stomatal water loss from the aerial parts of terrestrial plants [52]. In this study, *OsPYL9* mutant plants showed increased cuticle wax on the adaxial side of leaves, chlorophyll content as well as enhanced survival, decreased transpiration rate, and stomatal conductance. Previous studies have revealed that wax biosynthesis-related genes generally confer enhanced plant drought tolerance by accumulating more wax, whereas plants with reduced wax accumulation, decreased chlorophyll content, and water loss resulted in enhanced drought sensitivity [47,48,49,50]. The drought tolerance of *OsPYL9* mutants was in agreement with their enhanced accumulation of leaf cuticular wax, implying its role in drought stress tolerance.

The transcriptional regulation of carotenoid biosynthesis plays a key role in leaf tissues and the mutant showed translucent and needle-like leaves and enhanced drought tolerance [53,54,55,56]. Previous studies showed that cutin synthesis and the transport of wax are closely associated. Leaves of higher plants consist of a cutin embedded cuticle having interacuticular waxes synthesized and secreted by epidermal cells and confer the prevention of uncontrolled nonstomatal water loss [57,58,59]. Circadian rhythm signaling pathways are actively involved in plant physiological processes. Photosynthesis, light, temperature, and respiration are major periodic changes that influence plant circadian rhythm. Multiple proteins are involved in this phenomenon and plants have an endogenous central oscillator that regulates many aspects of circadian rhythm [60]. Homologous recombination (HR) protein pathways were also enriched, which supported the previous hypothesis that the CRISPR/Cas9 generated double-stranded break (DSB) in the target DNA is subsequently repaired by cell’s natural repair mechanism of HR or non-homologous end joining (NHEJ) [61]. The GO analysis showed that DEPs related to abiotic stimulus and ABA signaling pathways were significantly regulated. KEGG analysis revealed that the DEPs were only up presented in circadian rhythm pathway and late elongated hypocotyl (LHY) and two regulatory subunits (CK2α and CK2β) of casein kinase II were upregulated. Late elongated hypocotyl (LHY) is a core component of the circadian oscillator in plants and proposed to be an interlocking network of proteins working in a feedback loop [62]. Casein kinase 2 (CK2) is a heterotetramer composed of two catalytic (CK2α) and two regulatory subunits (CK2β) and an evolutionary and essentially conserved Ser/Thr protein kinase [63]. In Arabidopsis genome, CK2 contains four α-subunits (αA/CKA1, αB/CKA2, αC/CKA3 and αcp) and four β-subunits (β1/CKB1, β2/CKB2, β3/CKB3 and β4/CKB4) [64]. According to reports, CK2 is a critical component of the circadian clock systems of various organisms and involved in various stress responses including drought, temperature, and hormonal response [65,66,67].

The results revealed that most of the circadian clock, drought responsive and ROS-related proteins were upregulated, which were related to the closing of stomata, and the enhancement of resistance-related metabolism in plants under stress. *GIGANTEA* (*OsGI*) is homologous to Arabidopsis *GIGANTEA(GI)* At1g22770 and a regulator of rice in response to osmotic stress [68]. Early flowering gene (*OsEF3*) regulates the heading date and may also affect root development and thousand-grain weight by prolonging cell division and cell elongation [69]. Days to heading 7 (*DTH7*) is a major genetic site that controls rice photoperiod sensitivity and grain yield. It encodes a pseudo-response regulator protein whose expression is regulated by photoperiod. *DTH7* is expressed constitutively and is expressed in roots, stems, and leaves, and its expression exhibits a clear circadian rhythm [70]. FLAVIN-BINDING, KELCH REPEAT, F-BOX 1 (*OsFKF1)* knockdown mutants showed a significantly reduced ear number, fertility, and grain weight per plant, and had a certain increase in the number of grains per ear [71].

Calcium-dependent protein kinase 13 (*OsCDPK13)* overexpression plants have significantly improved tolerance to high salt, cold and drought stress [72]. Previous studies found that *OsCIPK* genes were differentially induced by polyethylene glycol (PEG), and ABA treatment, and by drought, salinity, cold stress [73]. WRKY transcription factor 30 (*OsWRKY30*) is activated by mitogen-activated protein kinase (MAPK), which imparts drought tolerance to rice. *OsWRKY30* interacts with *OsMPK3*, *OsMPK4*, *OsMPK7*, *OsMPK14*, *OsMPK20-4* and *OsMPK20-5*, and is phosphorylated by *OsMPK3*, *OsMPK7*, and *OsMPK14* [74]. The DREB and CBF transcription factors play an important role in plant development and abiotic stress responses. Plants over-expressing AP2/EREBP transcription factor (*OsDREB2A*) were found to have improved survival rates under severe salt and drought stress conditions. The ABA responsive element binding factor 1 (*OsABF1*), is an ABA response element binding factor, has transcriptional activation activity, and participates in abiotic stress response and ABA signaling in rice [75,76]. SALT-AND DROUGHT-INDUCED RING FINGER 1 (*OsSDIR1*) protein, which can be used as a candidate drought tolerance gene in crop genetic engineering and *OsSDIR1* transgenic show stronger drought tolerance [77]. NAC protein (*OsNAC10*/*ONAC122*) belongs to NAC transcription factor and its specific expression enlarges the root system and enhances drought tolerance, thereby increasing rice yield under drought [78]. DST is a new type of zinc finger transcription factor, which has a regulatory effect on drought and salt tolerance in rice. It can directly regulate the expression of *Gn1a/OsCKX2* to increase rice grain yield [79]. Dehydration-responsive element-binding protein 1A (*OsDREB1A*) specifically binds to drought response element/C repeat cis-element (DRE/CRT) and controls the expression of many stress-induced genes. In Arabidopsis, the overexpression of *OsDREB1A* can induce the expression of stress-related genes and improve the tolerance to drought, high salt, and cold stress. Therefore, *OsDREB1A* has the potential to cultivate drought-tolerant, salt-tolerant, and cold-tolerant transgenic rice [80].

In short, drought-tolerant rice plants have a close relationship among the expression of rice circadian clock genes and stress-responsive genes. The upregulation of rice drought stress-responsive and circadian clock genes together showed that there may be a regulatory relationship between the expression of these genes. Therefore, this study believes that the regulatory relationship between the expression of cell dehydration-responsive genes and the circadian clock genes optimize the water stress response at different times of the day. This mechanism plays an important role in increasing the survival rate and production efficiency of rice in the arid environment. In-depth research on the elements of this mechanism will contribute to the development of molecular breeding strategies and will also be of great significance to improve rice drought tolerance and increasing agricultural yields. Previous studies have shown that the PRR5-VP protein improves Arabidopsis biomass, and play a great role in cold, drought, and salt stress tolerance [81]. The *PRR37* polymorphism provides a breeding option for rice high-yield, plant type, heading date, and other trait resources [82]. The *OsPRR37* gene sequence is polymorphic in natural varieties, which greatly expands the latitude of rice planting, from the tropics to the sub-tropic zone, and the growth period varies widely. The biological clock plays a vital role in enhancing the adaptability and resistance of plants in the agricultural field, transforming the geographical limits of crops and increasing biomass [83]. Although there have been many studies on the circadian clock rhythms, the complete and complex signal network between the circadian rhythm and the stress signal has not been fully studied. It is far from enough to explore the functions of plant circadian clock genes, especially the related research on rice. However, how changes in the expression of circadian clock genes affect the expression of these stress resistance genes needs further research. The developed mutants in this study will provide the basic material to identify the interaction mechanism between circadian clock genes and drought response genes and explore the possibility of increasing the rice yield and enhancing rice adaptability under water deficit conditions.

This study provides a unique molecular mechanism underlying rice drought tolerance. According to our knowledge, previously many studies have been found on drought-tolerant mutants but there is no study that exists in which mutants were generated by CRISPR/Cas9 technology and proteome-wide alterations assessed in response to *OsPYL9* mutations. Previous studies are mostly based on map-based cloning which can provide the data on only a single gene, whereas the combination of CRISPR/Cas9 and proteomics technologies together opened the new way to comprehend the effect of single gene mutation proteome wide. Further studies are necessary to explore the molecular regulatory mechanism underlying the circadian clock and drought responsive genes to devise strategies for improving the rice phenotype. The mutants produced in this study are usually more valuable to farmers and have worthwhile advantages to plant breeders to produce new and better cultivars by employing modern technologies.

## 4. Materials and Methods

### 4.1. Experimental Material

Seeds of IR-96 rice variety were collected from Dr. Rongbai Li, Wild Rice Lab, Guangxi University (GXU), Nanning, China. Matured rice seeds were used as explants for Agrobacterium-mediated transformation of CRISPR/Cas9 construct. The pYLCRISPR/Cas9PubiH vector and promoters (OsU6a and OsU6b) were given by Dr. Yaoguang Liu’s lab, South China Agricultural University, Guangzhou, China. Plants were grown in a standard greenhouse conditions, and in the experimental field of Guangxi University (22° N, 108° E), Guangxi Province, China.

### 4.2. Targets Selection, Generation of the Guiding RNAs Expression Cassettes and Construction of CRISPR-Cas9 Binary Vector

**Figure 9:**
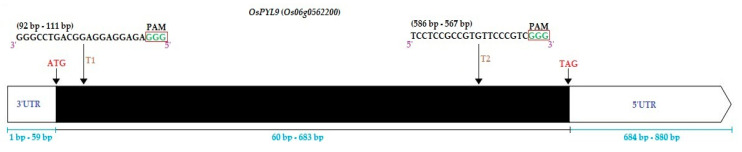
Gene structure and single guided RNAs (sgRNAs) positions of OsPYL9. T1 and T2 represent Target1 and Target2, respectively. The white boxes at the top left and right represent 3′UTR (untranslated region) and 5′UTR regions, respectively. The black box represents the exon region. PAM; protospacer adjacent motif, ATG, and TAG represents the start and stop codons, respectively.

The basic CRISPR-Cas9 binary vector was generated as described previously [84]. Target sequences were selected in the exon region of *OsPYL9* (Target1; 92 bp–111 bp, Target2; 586 bp–567 bp) using online tool CRISPR-GE (http://skl.scau.edu.cn/) (Table S7; Figure 9). The gRNA target oligonucleotides were synthesized by BGI (Beijing Genomics Institute). The secondary structures of both sgRNAs were developed by using the CRISPR-P ver 2.0 online tool (http://crispr.hzau.edu.cn/CRISPR2/) (Figure S4). The annealing products of target-sense and target-anti-sense oligonucleotides of sgRNA1 and sgRNA2 were ligated with OsU6a OsU6b, respectively, by using specific primers (Table S1). The PCR product of both samples was combined and purified by *TaKaRa MiniBEST* *Purification Kit* Ver.4.0. The expression cassette was pooled, and the restriction-ligation reaction was set up with pYLCRISPR/Cas9 plasmid. This construct was transformed into Agrobacterium tumefaciens strain EHA105 and used for the rice transformation. The CRISPR-Cas9 binary vector was created and confirmed as previously described [84]. Five potential off-targets containing at least a single nucleotide mismatch for both sgRNAs were selected for off-target analysis using the CRISPR-GE by default parameters (Table S4).

### 4.3. Rice Transformation and Genotyping of Mutant Plants

Healthy seeds of IR-96 were manually de-husked and washed with ddH_2_O three times. Surface sterilization of seeds was performed with 70% ethanol 4–5 times followed by 90 s with sterile ddH_2_O. Seeds were gently shaken for 20 min after sterilizing them with 50% (*v*/*v*) commercial bleach and washed with ddH_2_O for six times. Seeds were kept on autoclaved Whatman paper (3 mm) for 1 h and twenty seeds were inoculated per plate on callus induction media (CIM) and incubated in dark conditions at 26 ± 2 °C. The sgRNA expression cassette was transformed into *A. tumefaciens* EHA105 by electroporation and the transformation experiment was performed following Hiei et al. (1994) [85]. The regenerated mutant plants were screened by using hygromycin selectable markers (HPT-F/R). The transgenic seedlings (T_0_) were rescued and further advanced to the T_1_ generation in Guangxi University field, and then stored at 4 °C. The genomic DNA of mutant plants was extracted following the cetyltrimethylammonium bromide (CTAB) method and genotyping was performed using target-specific primers (OsPYL9F/R) (Table S1). T-DNA-free lines were obtained using Cas9 and HPT primers (Cas9F/R; HPTF/R), and iTRAQ-based proteomic analysis (Table S1). The sequencing results were viewed using the DSDecode M tool [86]. DNA and protein sequences alignments were carried out using the Clustal V method [87], with the default parameters.

### 4.4. Drought Assays at Seedling and Mature Stage

Seeds of WT and mutant lines were grown under the controlled conditions (16 h light at 28 °C and 8 h dark at 20 °C with 80% humidity) in a growth chamber. Rice seedlings of WT and mutant lines at the three-leaf stage (after five weeks) were subjected to the 20% PEG4000 hydroponic nutrient solution to simulate the drought stress. Leaf tissues were stored in a refrigerator at −80 °C for further analysis. For the drought assays of mature rice plants at Guangxi University field (18°25′ N, 108°58′ E), five transgenic plants from each line were tested. The germinated seeds were planted in a seedbed field. At the 3-leaf stage, the seedlings were transplanted into the testing field, with 3 replicates planted in the same block. The WT seedlings were also planted in the same block and used as the control. Watering was stopped at the panicle initiation stage to give drought stress at flowering and grain-filling stages. Meanwhile, WT and the same mutant plants were also grown under normal watering conditions. The agronomic data were recorded for PH, PN, PL, FLL, FLW, GNPP, GWT, GL, GWD, and YPP at the rice maturation stage.

### 4.5. Biochemical Assays under Normal Conditions and Drought Stress

The stressed and unstressed fresh rice seedlings (0.2 g) were crushed into a powder in a mortar with a pestle using LN_2_ (liquid nitrogen) and shaken overnight after homogenizing in 1 mL ddH_2_O. The sample was centrifuged, and the supernatant was used for the ABA assay. The ABA level was determined using radioimmunoassay (RIA) [88]. The crude enzyme from the powder was extracted in 50 mM chilled sodium phosphate (Na₃PO₄) buffer (pH 7.8) and 1% (*w*/*v*) polyvinylpolypyrrolidone (PVPP) at 4 °C. Centrifugation of the homogenate was performed for 15 min at 12,000× *g* and 4 °C. The MDA contents were determined by following the previously established method [88], and results were expressed as mg·g^−1^ fresh weight (FW) of the seedlings. In the supernatant, SOD, CAT, and POD enzyme activity was immediately determined. The same homogenized supernatant was used to analyze the protein content of the tissue for the calculation of specific enzyme activity. Normalization was performed by total FW to analyze the activity of an enzyme. Protein activity was determined using the Bradford protein assay kit (Sangon Biotech, Shanghai, China). The SOD activity was determined by the nitro blue tetrazolium (NBT) photochemical reduction by 50% according to the established protocol [89]. The reaction mixture contained 2.25 mM nitrotetrazolium blue chloride (NBT), 390 mM methionine, 3 mM EDTA, 150 µL crude enzyme extract, and 1.5 M Na_2_CO_3_. The reaction pH was adjusted to 10.2 and it was initiated by adding 1 mL 60 μM riboflavin. The solution was placed below a light source of 18 W fluorescent lamps for 10 min and absorbance was recorded at 560 nm with a UV–visible spectrophotometer (Model Bio Mate 3, Thermo Electron, Waltham, MA, USA). The quantity of SOD generated the 50% reduction of NBT in the reaction and was defined as one unit of enzyme activity. Specific enzyme activity was expressed as units per milligram of protein. CAT activity was measured by the rate of H_2_O_2_ decomposition at ΔA240 nm with a UV–visible spectrophotometer. The decomposition of H_2_O_2_ was determined after the absorbance decreased at 240 nm. Activity was calculated as the H_2_O_2_ extinction coefficient of 36 μM-1 cm^−1^ and units were indicated as min^−1^ g^−1^ FW, following the previously established method [90]. POD activity was assayed according to the previously established method [90]. The reaction mixture contained 0.1 M sodium phosphate buffer (pH 6.0), 9 mM guaiacol, and 2 mM H_2_O_2_. The reaction was started by adding 50 μL crude enzyme extract. Absorbance was measured at 470 nm with a UV–visible spectrophotometer. The activity was calculated as the H_2_O_2_ extinction coefficient of 6.39 μM-1 cm^−1^ and units were expressed as min^−1^ g^−1^ FW. Sixty day-old plants were used to measure stomatal conductance and transpiration rate during sunny days (09:00 a.m., 11:00 a.m., and 15:00 p.m.) by using the Li-Cor 6400 Portable Photosynthesis System. Chlorophyll was extracted from 0.1 g leaf samples with 80% acetone and measured according to the previous method, as explained by Lichtenthaler (1987) [91]. Olympus CX40 microscope was used to observe the leaves of WT and mutant plants at tillering stage and photographed with mshot-mc50.

### 4.6. Protein Extraction, Digestion, and Labelling

Protein extraction was performed according to Wang et al. (2014) [92], from 100 mg leaf samples of WT and mutant lines (GXU16-1-1, GXU16-2-1, GXU16-3-1, GXU16-4-1, GXU16-5-1, GXU16-6-1, GXU16-7-1, GXU16-8-1, GXU16-9-1, GXU16-10-1, GXU16-11-1, GXU16-12-1, GXU16-13-1, GXU16-14-1, and GXU16-15-1) grown under normal conditions. Three independent replicates were used for GXU16-9-1, whereas no replicate was used for the other mutants. Leaf samples were ground to a fine powder using liquid nitrogen (LN_2_) and lysis buffer (100 mM NH4HCO3(pH = 8), 6 M urea, and 0.2% SDS) was added and centrifuged for 15 min at 4 °C. The supernatant was removed, and 20 mL pre-cooled acetone was used to wash the pellet three times. Dissolution buffer containing 0.1 M triethylammonium bicarbonate (TEAB, pH = 8.5) and 6 M urea was used to dissolve the pellet. Bradford protein assay was used for the determination of protein concentration and 0.12 mg protein from each sample was digested by Trypsin Gold (Promega) at 1:50 enzyme-to-substrate ratio for 16 h at 37 °C, and vacuum dried. iTRAQ^®^ Reagent-8PLEX Multiplex Kit was used to label the peptides according to the manufacturer instructions. HPLC (high-performance liquid chromatography) fractionation and LC–MS/MS analysis were carried out by following a previously established procedure [92]. The iTRAQ-labeled peptides were fractionated at a high pH reverse phase separation to increase proteomic depth. The peptides were resuspended with loading buffer (2% mM HCOONH4, 2 M NaOH, pH10), separated by high pH reversed-phase liquid chromatography (Thermo SCIENTIFIC Vanquish F UHPLC). The gradient elution was conducted on high pH UPLC column (ACQUITY UPLC BEH C18 Column 1.7 μm, 2.1 mm × 150 mm, Waters, USA) at 200 μL/min with the gradient increased for 56 min (Phase B: 20 mM HCOONH4, 2 M NaOH, 80% acetonitrile ACN, pH10). Experiments were completed on an Orbitrap Fusion mass spectrometer that was coupled with an Easy-nLC 1000. Each of fractions was injected for nano LC–MS/MS analysis. The peptide mixture (2 μg) was loaded onto a C18 column (75μm × 25 cm, Thermo, Waltham, MA, USA) in buffer A (2% C_2_H_3_N and 0.1% CH_2_O_2_) and separated with a linear gradient of buffer B (90% C_2_H_3_N and 0.1% CH_2_O_2_) with a flow rate of 300 nL/min. The electrospray voltage of 1.8 kV versus the inlet of the mass spectrometer was used. Orbitrap Fusion mass spectrometer functioned in the data-dependent mode to switch automatically between MS and MS/MS acquisition. Survey full-scan MS spectra (400–1600 m/z) were attained through a mass resolution of 60 K, followed by twenty sequential HCD (high energy collisional dissociation) MS/MS scans with a resolution of 15 K. One micro scan was recorded using a dynamic exclusion of 25 s in each case. Normalized collision energy for MS/MS, was set at 30.

### 4.7. Proteomic Data Analysis

The sequence of the Cas9 protein (Q99ZW2) and HPT proteins (G0FGT3) was retrieved from Uniprot database to analyze the T-DNA-free plants. Proteome Discoverer 2.1 (Thermo Fisher Scientific) against *Oryza sativa* subsp. indica (Rice) database (17 September 2018; 40,869 entries) with default parameters was used to analyze the proteomic data. The highest score for a given peptide mass (best match to that predicted in the database) was used to identify the parent proteins. The parameters for protein searching were set as follows: trypsin digestion with up to two missed cleavages, the carbamidomethylation of cysteines as fixed modification, and the oxidation of methionines, and the protein N-terminal acetylation as variable modifications. Peptide spectral matches were validated, and the FDR verification was performed to remove peptides and proteins with FDR > 1%. The mass spectrometry results of GXU16-9-1 were further analyzed to find DEPs. The identified DEPs were annotated using the GO database (http://www.geneontology.org/) and the KEGG database (http://www.genome.jp/kegg/pathway). Fisher’s Exact Test was used to identify enriched GO terms and the Cluster 3.0 software used to perform cluster analysis of the differentially accumulated proteins (DAPs). The FC was calculated by the difference between the quantitative value of protein abundance in both samples. Proteins were quantified as a change in relative expression with adjusted FDR; proteins with FC ≥ 1.50 (*p* < 0.05) were considered upregulated and those with FC ≤ 0.67 (*p* ≤ 0.05) as downregulated between WT and mutants. The two-tailed Fisher’s exact test was employed to test the enrichment of the differentially expressed protein against all identified proteins. Correction for multiple hypothesis testing was carried out using the standard FDR control methods. The purpose of performing FDR correction is to reduce the Type-1 error by Bonferroni correction (multiple hypothesis test method). The GO or pathway with a corrected *p*-value ≤ 0.05 was considered significant. FDR thresholds for all stages were adjusted. STRING database (version 10.0) (https://string-db.org/) was searched for PPI network for all up and downregulated proteins. The network was then visualized by Cytoscape version 3.8.0.

### 4.8. RT-qPCR Analyses

The RT-qPCR analyses were conducted as previously described [93]. We also performed the RT-qPCR to validate the proteomic data. Each reaction was run in three replicates and rice *Actin* gene used as an internal control to normalize the data. Primers used were designed by using an online tool (https://biodb.swu.edu.cn/qprimerdb/) (Table S8) and the gene expression calculated by using 2^−ΔΔ^CT (cycle threshold) process as described previously [94].

### 4.9. Statistical Analyses

Statistical analysis (*p* < 0.05) was performed using SPSS 16.0 Statistical Software Program and GraphPad Prism (version 7.0, GraphPad Software Inc., San Diego, CA, USA) was used to develop the graphs.

## 5. Conclusions

The present study reported that *OsPYL9* mutagenesis confers drought tolerance by increasing enzymatic activities, wax accumulation, and upregulating proteins related to abiotic stress, circadian rhythm, and ROS activities. The CRISPR-guided mutagenesis of OsPYL9 has great potential for improving drought resistance and the yield of rice together. The availability of these mutant plants will be beneficial for further studies of the molecular mechanism of waxes’ responses in rice to environmental stresses. *OsPYL9* mutations directly influence the pathways related to abiotic stress and the expression of many stress responsive proteins. Additional work with these mutants will help us to gain a better understanding of the function of *OsPYL9*. The relationship between the upregulation of the circadian clock and drought responsive proteins will provide a sharp focus in the molecular breeding of rice. Undeniably, heritable homozygous mutants lacking T-DNA and off-target effects will be utilized as a basic material for future breeding programs. Functional analysis of the ABA receptor gene family will help to a design climate-resilient rice crop with “*more crop per drop*” and multiple abiotic stress tolerance.

## Figures and Tables

**Table 1:** Enhanced grain yields of the mutant rice plants in T_0_ and T_1_ generation under drought and well watered field conditions.

Treatment	Genotypes	PH	PN	PL	FLL	FLW	GNPP	GWT	GL	GWD	YPP
Normal (T_0_)	WT	119.3 ± 3.4	7.4 ± 1.6	26.3 ± 1.3	53.1 ± 2.2	2.1 ± 0.3	136 ± 09	29.4 ± 1.3	9.2 ± 0.3	3.1 ± 0.3	30.8 ± 2.5
	GXU16-1	123.5 ± 3.8 ^ns^	7.6 ± 1.5 ^ns^	26.6 ± 1.6 ^ns^	39.7 ± 3.3 *	1.6 ± 0.3 *	141 ± 10 ^ns^	37.8 ± 1.3 *	11.2 ± 0.2 *	3.6 ± 0.2 *	38.7 ± 1.7 *
	GXU16-2	124.6 ± 3.6 ^ns^	7.8 ± 2.2 ^ns^	26.5 ± 1.6 ^ns^	41.0 ± 2.5 *	1.5 ± 0.3 *	142 ± 11 ^ns^	38.4 ± 1.2 *	11.3 ± 0.3 *	3.7 ± 0.2 *	38.9 ± 1.4 *
	GXU16-9	125.4 ± 4.3 ^ns^	7.7 ± 1.4 ^ns^	25.9 ± 1.7 ^ns^	38.4 ± 1.6 *	1.5 ± 0.2 *	142 ± 11 ^ns^	38.6 ± 1.4 *	11.4 ± 0.5 *	3.7 ± 0.4 *	40.6 ± 1.5 *
Drought (T_0_)	WT	107.3 ± 3.2	7.7 ± 1.9	19.6 ± 1.5	47.2 ± 2.3	2.0 ± 0.3	84 ± 14	22.8 ± 1.2	7.1 ± 0.4	2.5 ± 0.2	21.4 ± 1.5
	GXU16-1	123.3 ± 3.3 *	7.7 ± 2.4 ^ns^	26.9 ± 1.6 *	37.8 ± 1.6 *	1.6 ± 0.4 *	112 ± 11 *	30.6 ± 1.3 *	8.8 ± 0.3 *	3.0 ± 0.2 *	26.8 ± 1.6 *
	GXU16-2	123.6 ± 4.2 *	7.6 ± 1.3 ^ns^	26.9 ± 1.2 *	39.3 ± 1.7 *	1.6 ± 0.2 *	113 ± 11 *	33.6 ± 1.2 *	9.5 ± 0.4 *	3.1 ± 0.4 *	31.6 ± 1.4 *
	GXU16-9	124.2 ± 4.1 *	7.9 ± 1.3 ^ns^	27.5 ± 1.4 *	36.4 ± 2.2 *	1.5 ± 0.3 *	115 ± 10 *	34.7 ± 1.6 *	10.1 ± 0.3 *	3.1 ± 0.3 *	32.6 ± 1.5 *
Normal (T_1_)	WT	1201 ± 2.4	7.6 ± 1.4	25.2 ± 1.2	52.0 ± 3.6	2.2 ± 0.1	137 ± 12	29.5 ± 1.2	9.0 ± 0.1	3.0 ± 0.2	31.3 ± 1.5
	GXU16-1-1	122.4 ± 3.6 ^ns^	7.5 ± 1.8 ^ns^	26.3 ± 1.4 ^ns^	39.5 ± 2.1 *	1.6 ± 0.2 *	140 ± 11 ^ns^	37.9 ± 1.5 *	11.0 ± 0.3 *	3.5 ± 0.1 *	38.5 ± 1.3 *
	GXU16-2-1	124.2 ± 3.4 ^ns^	7.7 ± 2.1 ^ns^	26.4 ± 1.3 ^ns^	40.3 ± 1.6 *	1.5 ± 0.4 *	139 ± 10 ^ns^	38.6 ± 1.3 *	11.1 ± 0.2 *	3.6 ± 0.3 *	39.1 ± 1.2 *
	GXU16-9-1	124.2 ± 5.4 ^ns^	7.8 ± 1.2 ^ns^	25.6 ± 1.2 ^ns^	38.5 ± 1.2 *	1.5 ± 0.3 *	140 ± 10 ^ns^	38.7 ± 1.1 *	11.3 ± 0.6 *	3.7 ± 0.5 *	40.2 ± 1.2 *
Drought (T_1_)	WT	108.1 ± 2.4	7.6 ± 1.7	19.3 ± 1.4	48.5 ± 2.6	2.0 ± 0.2	85 ± 15	23.5 ± 1.3	7.0 ± 0.3	2.6 ± 0.1	21.2 ± 1.0
	GXU16-1-1	125.2 ± 3.1 *	7.6 ± 2.1 ^ns^	26.4 ± 1.3 *	38.2 ± 1.9 *	1.6 ± 0.6 *	111 ± 10 *	29.4 ± 1.2 *	8.6 ± 0.2 *	3.0 ± 0.3 *	26.2 ± 1.4 *
	GXU16-2-1	124.1 ± 5.1 *	7.7 ± 1.0 ^ns^	27.4 ± 1.3 *	39.5 ± 1.4 *	1.6 ± 0.3 *	112 ± 10 *	34.5 ± 1.3 *	9.3 ± 0.3 *	3.1 ± 0.5 *	31.2 ± 1.2 *
	GXU16-9-1	123.1 ± 3.3 *	7.7 ± 1.2 ^ns^	27.6 ± 1.5 *	36.0 ± 3.6 *	1.5 ± 0.1 *	114 ± 12 *	34.5 ± 1.1 *	10.0 ± 0.2 *	3.1 ± 0.2 *	32.1 ± 1.3 *

WT (wild type); PH (plant height) cm; PN (panicle numbers); PL (panicle length) cm; FLL (flag leaf length) cm; FLW (flag leaf width) cm; GNPP (grain number per panicle); GWT (1000-grain weight) g; GL (grain length) mm; GWD (grain width) mm; YPP (yield per plant) g. Data are the mean of five plants from three independent replicates (*n* = 5). * and ^ns^ represent the significant and non-significant differences (Student’s *t*-test, *p* < 0.01), respectively.

**Table 2:** Some important differentially expressed proteins (DEPs) related to the abiotic stress response.

Proteins	Annotation	Regulation	Fold Change
	**Circadian Clock DEPs**		
Q9AWL7	GIGANTEA	Up	3.59
Q657D6	Early flowering protein	Up	1.62
Q689G9	Pseudo-response regulator 1	Up	1.55
A0A0N7KPA9	MYB family transcription factor	Up	1.56
Q0D3B6	Days to heading 7	Up	1.96
Q2R2W1	FLAVIN-BINDING, KELCH REPEAT, F-BOX 1	Up	1.77
C6F1N5	Pseudo-response regulator 59	Up	3.36
Q689G6	Pseudo-response regulator 95	Up	1.70
	**Drought Responsive DEPs**		
Q9FXQ3	Calcium-dependent protein kinase 13	Up	2.08
Q5JLS2	CBL-interacting protein kinase 12	Up	1.68
Q6ZKN0	WRKY transcription factor 30	Up	1.57
Q0JQF7	AP2/EREBP transcription factor	Up	1.81
Q0JHF1	ABA responsive element binding factor 1	Up	2.29
Q10NE1	SALT-AND DROUGHT-INDUCED RING FINGER 1	Up	1.56
Q0IUZ3	NAC protein	Up	2.78
Q0DMY6	DROUGHT AND SALT TOLERANCE	Up	2.15
Q64MA1	Dehydration-responsive element-binding protein 1A	Up	3.16
Q6IEN1	WRKY transcription factor	Up	1.87
	**DEPs Related to Reactive Oxygen Species**		
A3REN3	Catalase (CAT)	Up	1.68
B8ARU3	Peroxidase (POD)	Up	1.91
A2YPX2	POD	Up	1.59
A2X2T0	POD	Up	1.83
B8A755	POD	Up	1.58
B8B5W7	POD	Up	1.99
A2X822	POD	Down	0.56
B8B3L5	POD	Up	1.62
B8ASV8	POD	Up	2.09
A2Z4F1	POD	Up	1.65
B8B653	POD	Up	1.61
B8B5W6	POD	Up	1.73
Q01MI9	POD	Down	0.50
A2WPA1	POD	Up	1.74
A2WNR8	POD	Up	1.50
B8APG3	POD	Up	1.92
B8ARU4	POD	Up	1.62
A2Z9R2	POD	Up	3.19
B8BM92	POD	Up	1.86
A2ZAQ7	POD	Up	1.55
B8AWM4	Superoxide dismutase (SOD)	Up	1.56
A2YY59	SOD	Up	2.08

## Supplementary Materials

Supplementary Materials can be found at https://www.mdpi.com/1422-0067/21/21/7854/s1.

## Abbreviations

CRISPR	Clustered regularly interspaced short palindromic repeats
Cas9	CRISPR-associated protein 9
ABA	Abscisic acid
MDA	Malondialdehyde
CAT	Catalase
POD	Peroxidase
SOD	Superoxide dismutase
ROS	Reactive oxygen species
iTRAQ	Isobaric tags for relative and absolute quantitation
DEPs	Differentially expressed proteins
KEGG	Kyoto Encyclopedia of Genes and Genomes
GO	Gene Ontology
PPI	Protein–protein interaction
