# Supplementary material for: Precise Editing of the OsPYL9 Gene by RNA-Guided Cas9 Nuclease Confers Enhanced Drought Tolerance and Grain Yield in Rice (Oryza sativa L.) by Regulating Circadian Rhythm and Abiotic Stress Responsive Proteins
Source: Int J Mol Sci. 2020 Oct 23;21(21):7854. doi: 10.3390/ijms21217854 (PMC7660227; doi:10.3390/ijms21217854)
Supplement: Supplementary file 1 [file ijms-21-07854-s001.zip › Supplementary file 1.docx]

**Table S1.** Primers list used for construction of vector and genotyping of mutant plants.

| **Primer name** | **Primer Sequence (5'-3')** |
| --- | --- |
| OsPYL9T | F: AGGTCGATCAGAAATCAATGG |
|  | R: CGGTACAAGCACTTCGTCC |
| gRT1: | GGCCTGACGGAGGAGGAGAgttttagagctagaaat |
| OsU6aT1: | TCTCCTCCTCCGTCAGGCCCggcagccaagccagca |
| gRT2: | TCCTCCGCCGTGTTCCCGTCgttttagagctagaaat |
| OsU6aT2: | GACGGGAACACGGCGGAGGACaacacaagcggcagc |
| U-F | CTCCGTTTTACCTGTGGAATCG |
| gR-R | CGGAGGAAAATTCCATCCAC |
| Pps-R | TTCAGA**ggtctc**T**ACCG**ACTAGTATGGAATCGGCAGCAAAGG |
| Pgs-2 | AGCGTG**ggtctc**G**tcag**ggTCCATCCACTCCAAGCTC |
| Pps-2 | TTCAGA**ggtctc**T**ctga**cacTGGAATCGGCAGCAAAGG |
| Pgs-L | AGCGTGGGTCTCGTCTTCACTCCATCCACTCCAAGCTC |
| PB-R | GCGCGCGGTCTCTACCGACGCGTATCC |
| PB-L | GCGCGCgGTCTCGCTCGACTAGTATGG |
| HPT | F: GTGCTTGACATTGGGGAGTT  R: ATTTGTGTACGCCCGACAGT |
| Cas9-F | CTGACGCTAACCTCGACAAG |
| Cas9-R | CCGATCTAGTAACATAGATGACACC |
| SP-L1 | GCGGTGTCATCTATGTTACTAG |
| SP-R | GCCTATACCAAGTTATTGCA |

**Table S2.** Mutations types in T_0_ events achieved by transformation with two CRISPR/Cas9 constructs.

| **Sr. No.** | **Genotype** | **Target 1** | **Target 2** |
| --- | --- | --- | --- |
| 1 | GXU16-1 | -3/- | -2/- |
| 2 | GXU16-2 | -9/-9 | -4/-4 |
| 3 | GXU16-3 | -4/-1 | -3/- |
| 4 | GXU16-4 | WT | WT |
| 5 | GXU16-5 | -2/WT | -1/+1 |
| 6 | GXU16-6 | WT | WT |
| 7 | GXU16-7 | -1/-3 | -3/- |
| 8 | GXU16-8 | WT | WT |
| 9 | GXU16-9 | -11/-11 | -9/-9 |
| 10 | GXU16-10 | WT | -2/-1 |
| 11 | GXU16-11 | -1, +1/- | -2/-2 |
| 12 | GXU16-12 | -3/-2 | -2/- |
| 13 | GXU16-13 | -1, +1/- | -3/-1 |
| 14 | GXU16-14 | WT | WT |
| 15 | GXU16-15 | -3/-3 | -1/- |

^_^: deletion, +: insertion. The numbers in front of the dashes indicate the number of nucleotides affected. Corresponding mutations in both alleles are distinguished by ‘/’.

**Table S3.** Primers designed for off-target sites evaluation.

| **Primer name** | **Primer Sequence (5'-3')** |
| --- | --- |
| POT1 | F: CTGATGATGAGGGGTGTGTG  R: CCCTTGGACAACTCCTTGAA |
| POT2 | F: CGGGATGTAGTCGGAGATGT  R: ACAGCCCAACTGAGAGGAGA |
| POT3 | F: GCTGCCTACATCAACGACAA  R: CCCTTGGAACACTCCTTGAA |
| POT4 | F: GCTTCGTCCTCAACCTCAAG  R: CGATGTACTGCAGGATGACC |
| POT5 | F: CGTCGTCCTCAACCTCAAGT  R: CGATGTACTGCACGATGACC |
| POT6 | F: CCTGCTCCTTCCACTGGTAT  R: GCCAGTCTCCCTCATCAACT |
| POT7 | F: AGGAGATGGGTGTGGCTCTA  R: GCCTTCATCTTCTCCACCTG |
| POT8 | F: GCTTCTGGAGGTTGAGCTTG  R: AACTACCGCTCCGTCACCT |
| POT9 | F: CGAAGCACCTCCCTGAATAG  R: ATCAGGCTCAACCTCCAGAA |
| POT10 | F: CGCCCTCATCTACCAACCTA  R: CTCTCCATCTTCGCTGTTCC |

**Table S4.** Mutations detection on five most likely putative off-target sites.

| **Target** | **NOPO** | **GL** | **Locus** | **Sequence** | **MMB** | **NPS** | **NPM** | **OTS** | **Region** |
| --- | --- | --- | --- | --- | --- | --- | --- | --- | --- |
| T1 | OT1 | Chr10: 20590275-20590300 | LOC_Os10g38580 | GGGCCT**C**ACGGAGGAGGAGA **AGG** | 4 | 30 | 0 | 0.688 | CDS |
|  | OT2 | Chr12: 2683466-2683488 | LOC_Os12g05820 | GGGCC**A**GACG**A**AGGAGGAG**G** **GGG** | 4 | 30 | 0 | 0.663 | CDS |
|  | OT3 | Chr10: 20575372-20575394 | LOC_Os10g38540 | GGGC**AA**GACGGAGGAGGAGA **GGG** | 4 | 30 | 0 | 0.495 | CDS |
|  | OT4 | Chr10: 20646351-20646373 | LOC_Os10g38780 | G**A**GC**GA**GACGGAGGAGGAGA **GGG** | 5 | 30 | 0 | 0.440 | CDS |
|  | OT5 | Chr10: 20634335-20634357 | LOC_Os10g38740 | G**A**GC**AA**GACGGAGGAGGAGA **GGG** | 4 | 30 | 0 | 0.419 | CDS |
| T2 | OT6 | Chr12: 26985847-26985869 | LOC_Os12g43510 | **C**CCTCC**A**C**G**G**G**GTTCCCGTC **GGG** | 3 | 30 | 0 | 0.212 | CDS |
|  | OT7 | Chr5: 24624200-24624222 | LOC_Os05g42100 | **G**CCTCCG**A**CGTGTTC**T**CGTC **GGG** | 4 | 30 | 0 | 0.191 | CDS |
|  | OT8 | Chr2: 7111206-7111228 | LOC_Os02g13330 | TCCTCC**T**C**G**GTGTTCCC**C**TC **GGG** | 3 | 30 | 0 | 0.168 | CDS |
|  | OT9 | Chr10: 22766952-22766974 | LOC_Os10g42280 | TC**G**TCC**T**C**G**GTGTT**G**CCGTC **GGG** | 3 | 30 | 0 | 0.009 | CDS |
|  | OT10 | Chr6: 27764894-27764916 | LOC_Os04g46860 | TCC**G**CCGCC**CC**GTTCCCGTC **GGC** | 3 | 30 | 0 | 0.004 | CDS |

Note: The protospacer adjacent motif (PAM) (NGG) is shown in green background. NOPO; name of putative off-target, GL; genomic location, MMB; mis-matching bases, NPS; number of plants screened, NPOM; number of plants with off-target mutations, OTS; off-target score. T1 and T2 represents target1 and target2.

**Table S5.** Segregation pattern of homozygous, mono-allelic heterozygous, and bi-allelic heterozygous mutations in T_1_ generation.

| **Targets** | **T_0_** | | | **T_1_** | | | | |
| --- | --- | --- | --- | --- | --- | --- | --- | --- |
|  | **Mutants** | **Zygosity** | **InDels** | **PT** | **WT** | **Bi** | **Homo** | **Hetero** |
| T1 | GXU16-9 | Hom | 9d/9d | 15 | 0 | 0 | 15(6d/6d) | 0 |
| T2 | GXU16-9 | Hom | 4d/4d | 15 | 0 | 0 | 15(6d/6d) | 0 |
| T1 | GXU16-1 | Mono Het | 3d/- | 42 | 11 | 0 | 21(3d/3d) | 10(WT/WT) |
| T2 | GXU16-1 | Mono Het | 2d/- | 42 | 13 | 0 | 20(2d/2d) | 9(WT/WT) |
| T1 | GXU16-3 | Bi Het | 4d/1d | 42 | 0 | 21(4d/1d) | 11(4d), 10(1d) | 0 |
| T2 | GXU16-5 | Bi Het | 1d/1i | 42 | 0 | 21(1d/1i) | 10(1d), 11(1i) |  |

PT; number of plants tested, WT; wild type, Bi Het; bi-allelic heterozygous, Homo; homozygous, Mono Het; mono-allelic heterozygous, Chi; chimeric. d: deletion, i: insertion and WT: wild type. The numbers in front of the letters indicate the number of nucleotides affected. Corresponding mutations in two alleles are distinguished by ‘/’.

**Table S6.** The significant enrichment pathways with differentially expressed proteins (DEPs).

| **Pathway** | **DEPs with**  **Pathway Annotation** | **All Proteins with Pathway**  **Annotation (1808)** | **p Value**  **≤0.05** | **Pathway ID** |
| --- | --- | --- | --- | --- |
| Ribosome biogenesis in eukaryotes | 3/70 | 11 | 0.000853 | osa03008 |
| Carotenoid biosynthesis | 3/70 | 11 | 0.006782 | osa00906 |
| Phenylpropanoid biosynthesis | 6/70 | 49 | 0.007899 | osa00940 |
| Cysteine and methionine metabolism | 5/70 | 45 | 0.020917 | osa00270 |
| Starch and sucrose metabolism | 6/70 | 62 | 0.021412 | osa00500 |
| Phagosome | 4/70 | 31 | 0.02359 | osa04145 |
| Circadian rhythm | 8/70 | 8 | 0.03287 | osa04712 |
| Carbon metabolism | 3/70 | 183 | 0.042691 | osa01200 |
| Homologous recombination | 2/70 | 10 | 0.04891 | osa03440 |

**Table S7.** Selected target positions with their GC content and potential off-target score.

| **Sr. No.** | **Target Sequence (5’-3’)** | **Position** | **Strand** | **GC %** | **Region** | **Off-target Score** | **On-target Score** |
| --- | --- | --- | --- | --- | --- | --- | --- |
| T1 | GGGCCTGACGGAGGAGGAGA**GGG** | 92-111 | + | 70.0 | CDS | 0.688 | 0.1359 |
| T2 | TCCTCCGCCGTGTTCCCGTC**GGG** | 586-567 | - | 70.0 | CDS | 0.267 | 0.0297 |

T1 and T2; represents the Target1 and Target2. Green highlighted are PAM regions.

**Table S8.** Primers designed for RT-qPCR analysis.

| **Gene ID** | **Forward Primer (3’-5’)** | **Reverse Primer (5’-3’)** |
| --- | --- | --- |
| *OsPYL9* | CAAGCTCAACCTCCAGATGC | TGGACGACGACGACGATTTA |
| *OsGI* | TGCTGACAAGGCCACACCATG | GGAGCAAGCCCCGTATCATGC |
| *OsFKF1* | GGTTGGCGGCACCAGGGTTC | CTCATCTTCGTCGGGCCTGC |
| *OsABF1* | CAGGAGGAGCAACATCAGAAAC | GATCTCGTGCTGACGTTTTCC |
| *OsNAC10* | TTCTCCTCGACGGCTCATCC | ATGGATGGCTCAGCAGATTG |
| *OsDST* | TCCCCGGCAAGCACCACCAC | GAGGCTCAAGTTGAGGTCGAG |
| *OsCIN1* | GCTCACTCGGTATCACAGGA | GGTCGATCCCAGTGTAGAGG |
| *OsSSIII-1* | AAGCTGAAAGGAGGAGGCAT | CTTGTTCTGCAGCCCTTTGT |
| *OsAlba1* | CTCGCCAAGGTTTGTCACTC | TCGAGACCAGGGATGAACAG |
| *OsCLPD1* | AGGCTGTTAGCCCTTGGAAT | TCCTCAATCAGGTGGGTGAC |
| *Osoxo4* | CCAGTTCAACGTTGGCAAGA | TAGTACCCGCCGGTGAATTT |
| *OsActin1* | TTCCTCATGCCATCCTGCGTCTG | GTCCCTTACAATTTCCCGTTCAGC |


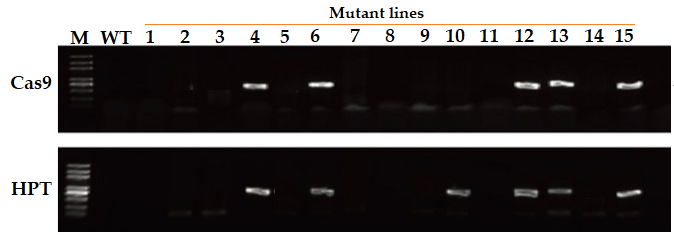


**Figure S1.** Screening of Transgene (T-DNA) free plants by polymerase chain reaction (PCR). WT represents wild type, whereas, 1-15 numbers represents mutant line from GXU16-1 to GXU16-15. Cas9; CRISPR associated proteins, HPT; hygromycin-B-phosphotransferase.

**
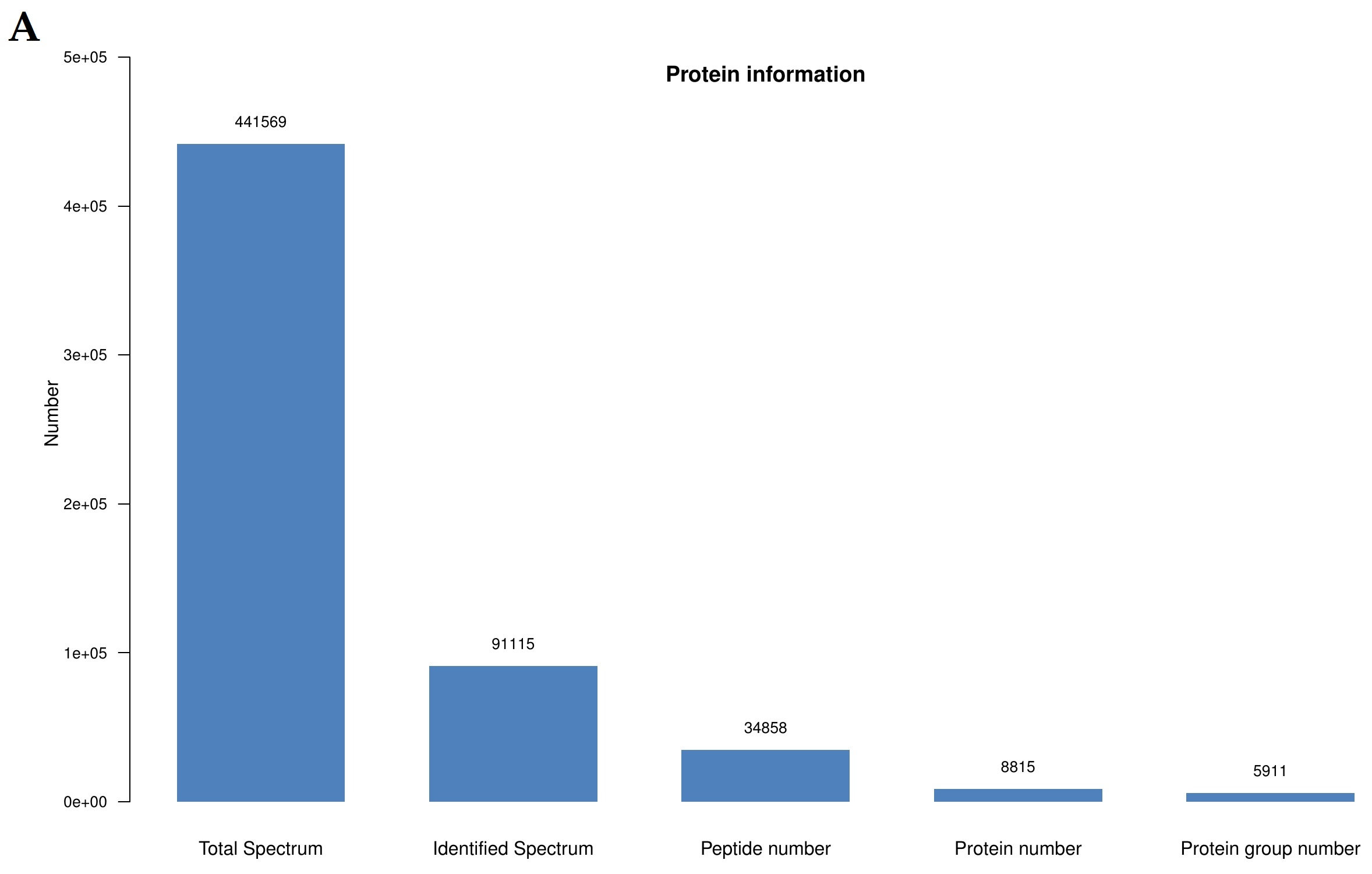

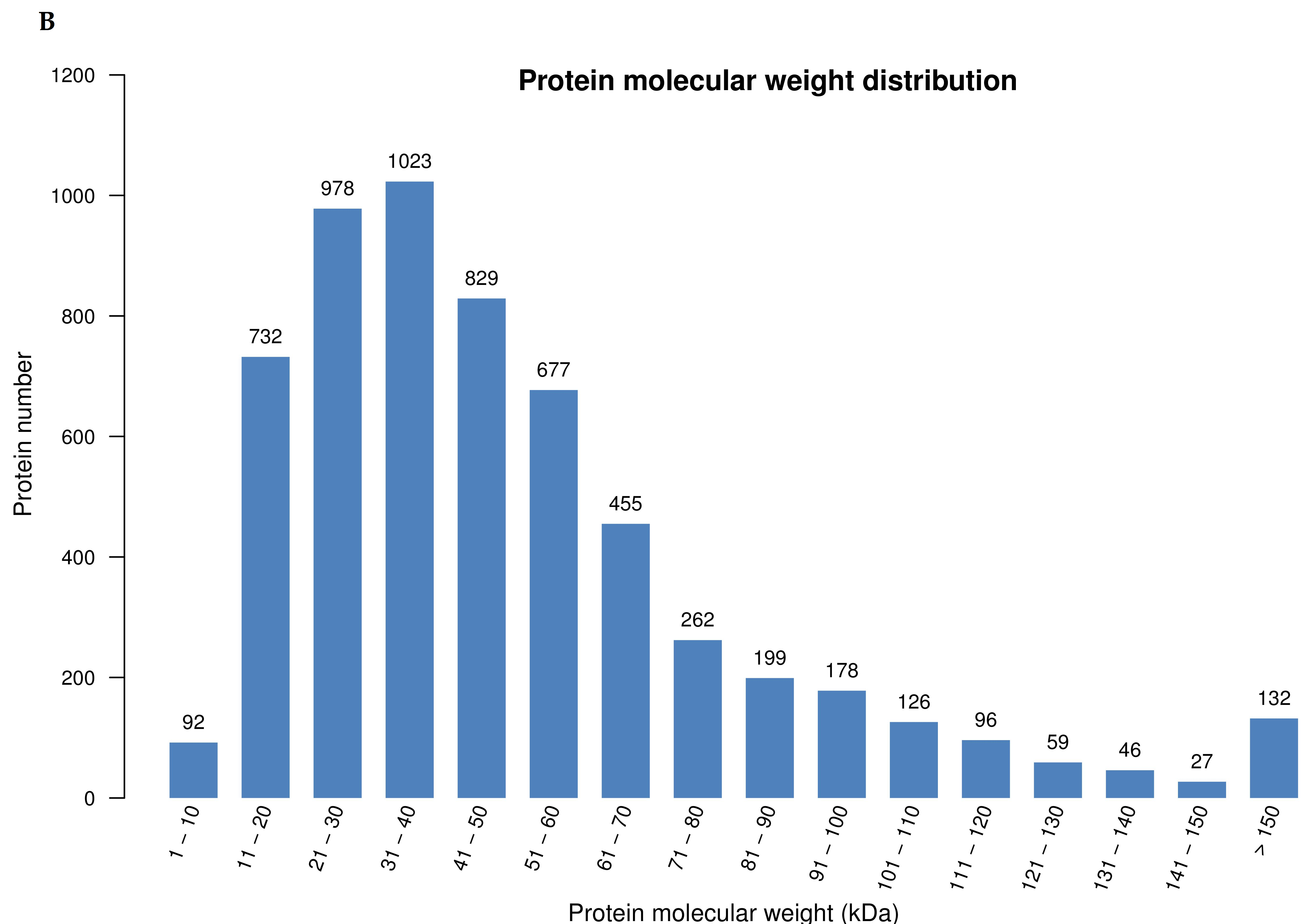

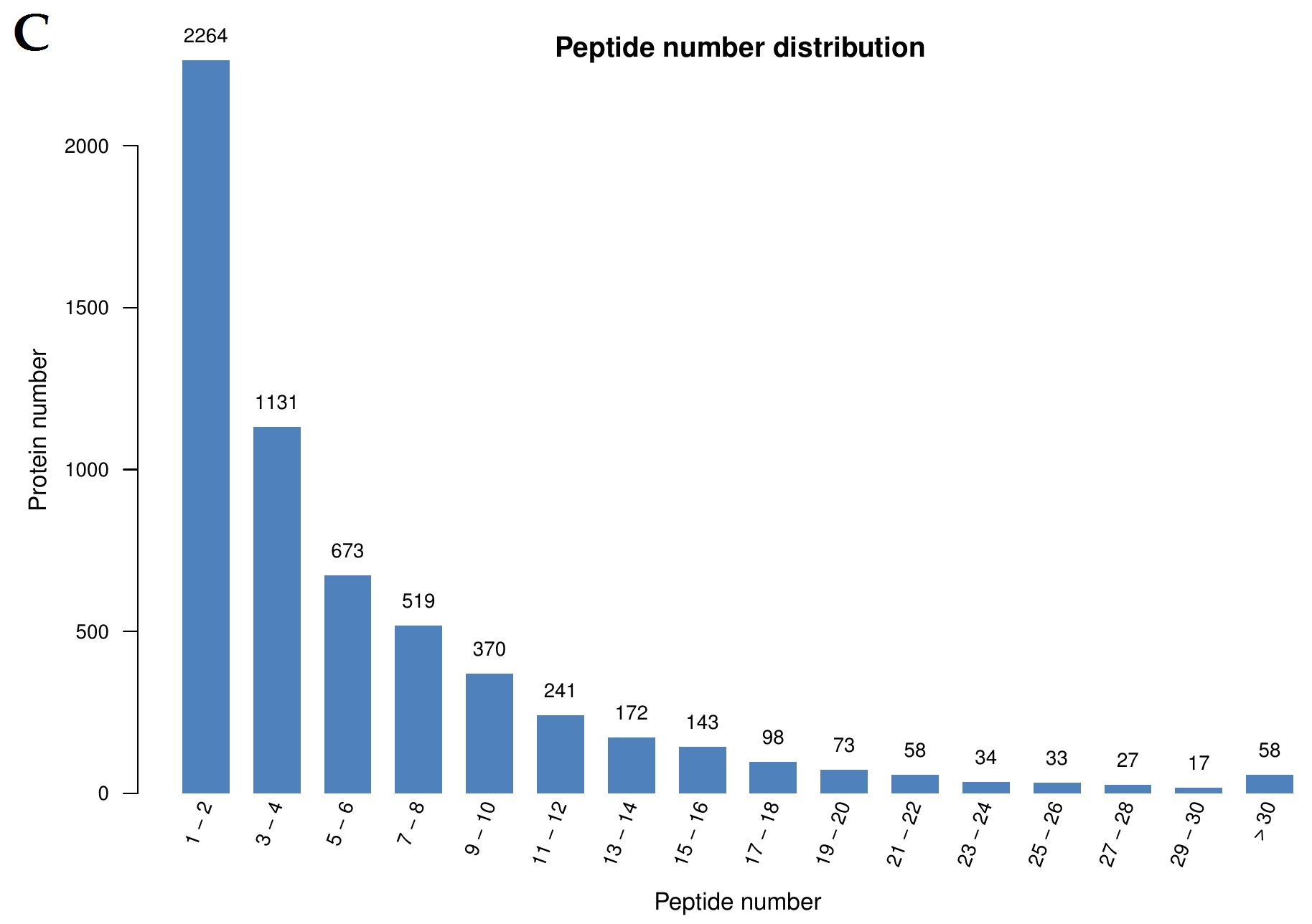

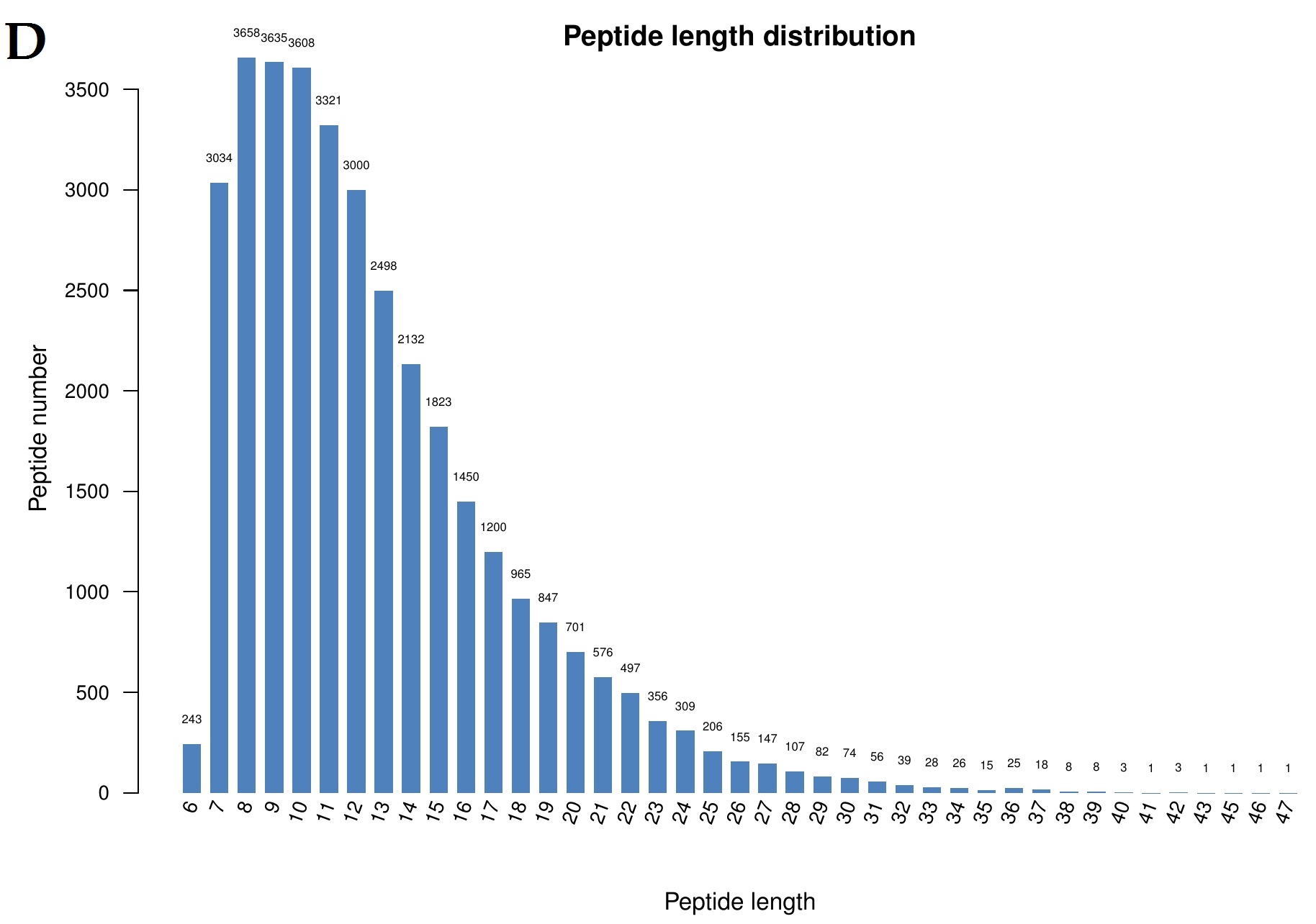
**

**
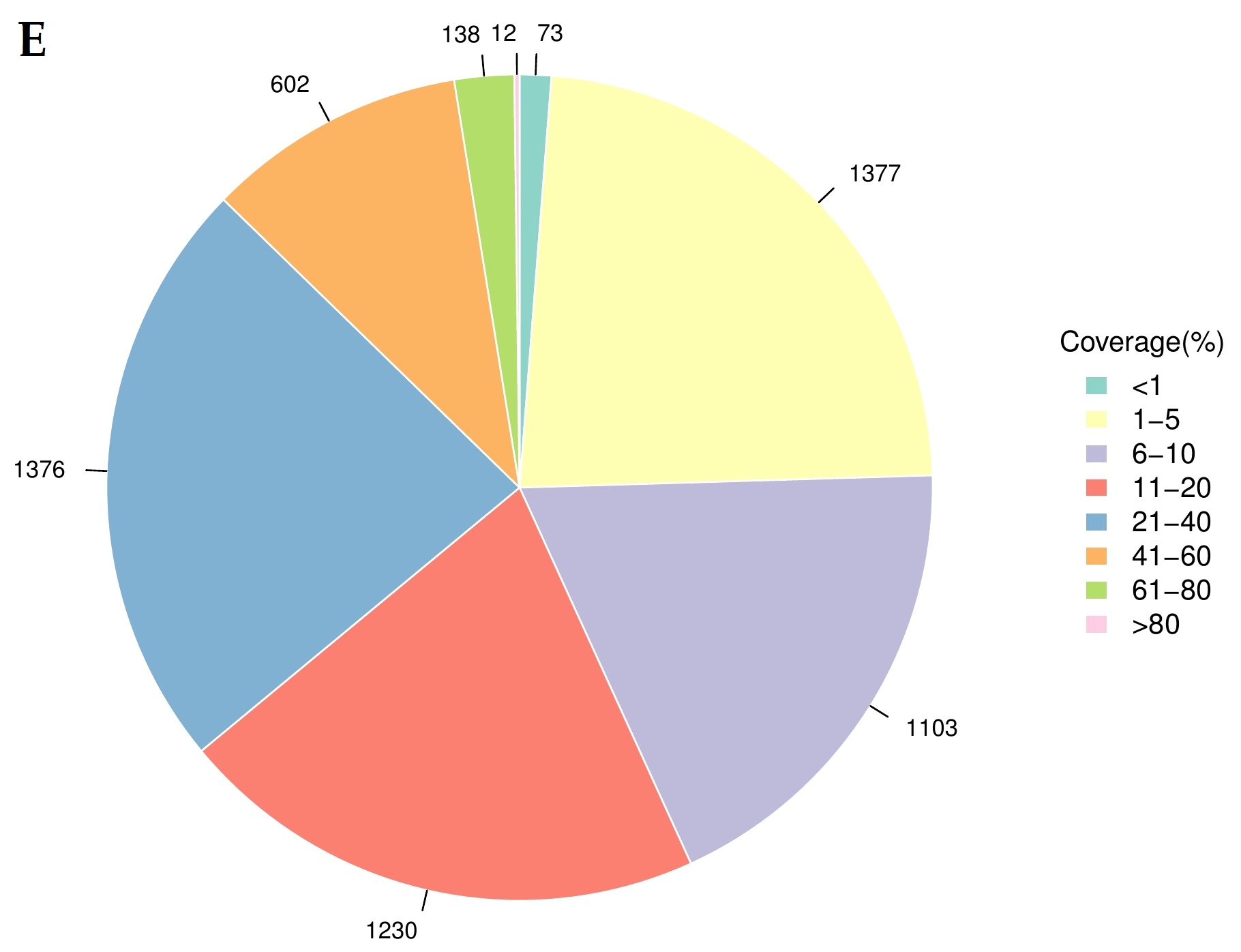

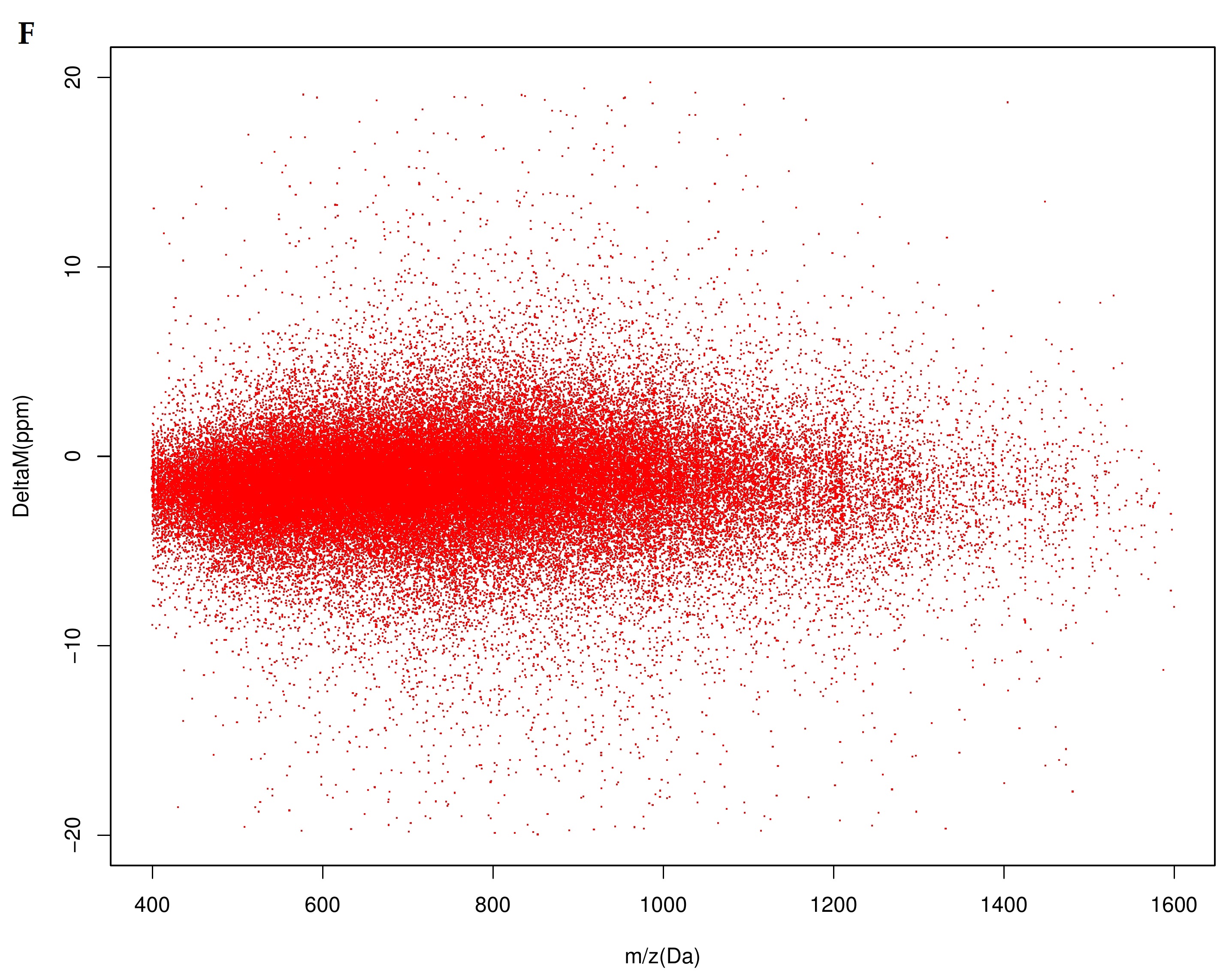
**

**Figure S2.** Basic information of identification and analysis of proteome in wild type and rice CRISPR mutant (GXU16-9-1) (**A**) Protein information histogram about total spectrum of MS/MS, the number of matched spectra, the peptide number, the protein number and the protein group number (**B**) Distribution of the identified proteins among the different molecular weight classes (unit: kilodalton, kDa) (**C**) Peptide number distribution histogram (**D**) Peptide length distribution. The x-axis indicates the peptide length (the number of amino acids) and the *y*-axis indicates the ratio of the peptide length to the total number of peptides **(E)** Protein sequence coverage distribution pie chart. The number outside the fan indicates the coverage of the protein number in this interval **(F)** Distribution of peptide segment matching error. X-axis is mass-to-charge ratio and Y-axis indicates the matching error of fragmented ions’ mass.

**
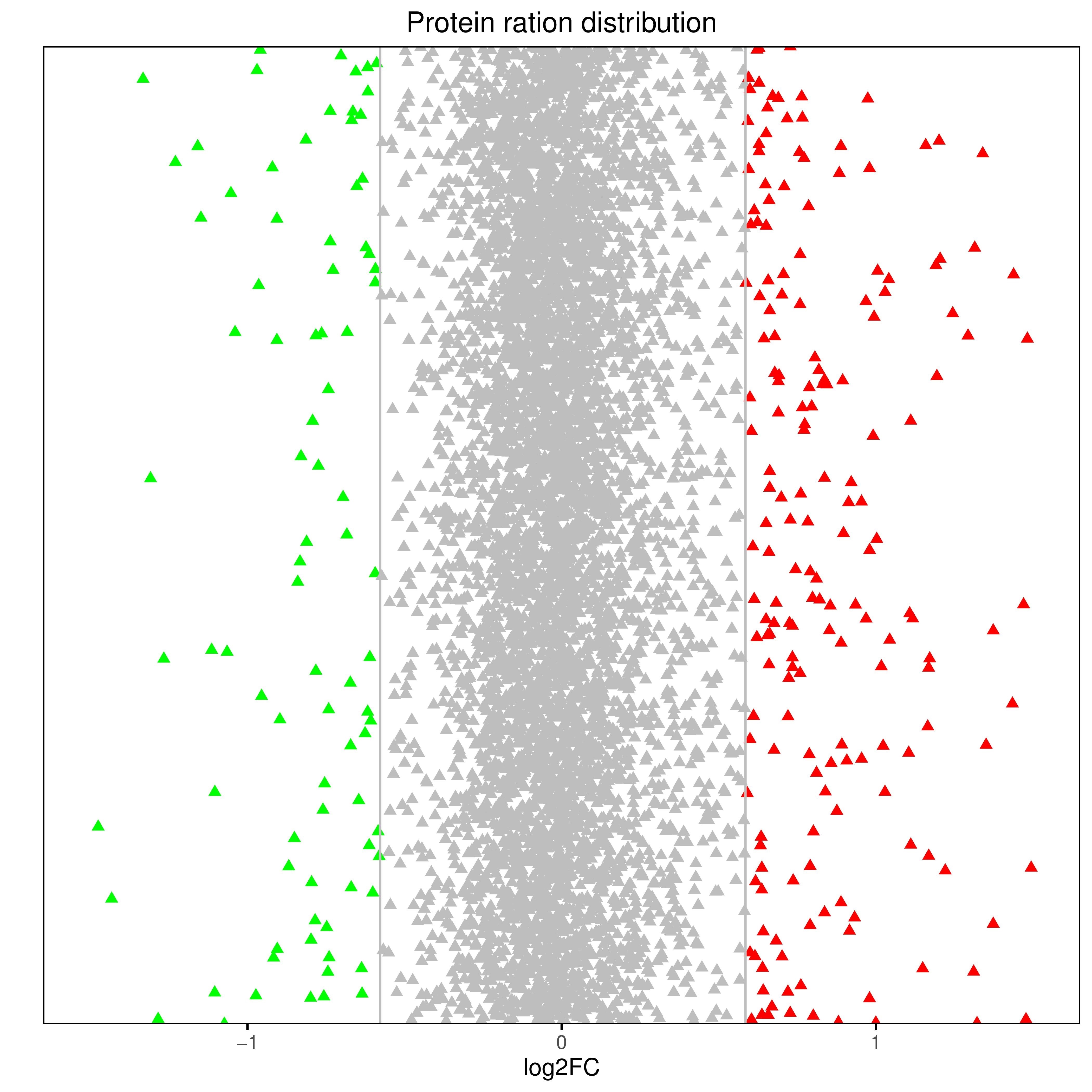
**

**Figure S3.** Scatter plots of the protein abundance distribution of expressed proteins. X-axis represents the fold change values between the two samples, based on a log2 scale. In the scatter plot, green and red dots represent significantly down-regulated and up-regulated proteins, respectively. The grey dots represent insignificant changed proteins.

**
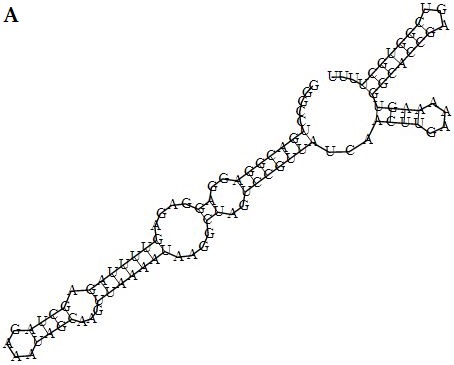

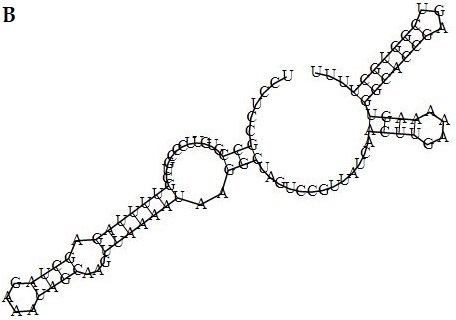
**

**Figure S4.** Schematic representation of secondary structures of **(A)** sgRNA1; and **(B)** sgRNA2; used in the experiment.
